# Child-to-adult body size change and risk of type 2 diabetes and cardiovascular disease

**DOI:** 10.1007/s00125-023-06058-4

**Published:** 2023-12-12

**Authors:** Germán D. Carrasquilla, Lars Ängquist, Thorkild I. A. Sørensen, Tuomas O. Kilpeläinen, Ruth J. F. Loos

**Affiliations:** 1 Novo Nordisk Foundation Center for Basic Metabolic Research, Faculty of Health and Medical Sciences, University of Copenhagen, Copenhagen, Denmark; 2 Department of Public Health, Section of Epidemiology, Faculty of Health and Medical Sciences, University of Copenhagen, Copenhagen, Denmark; 3 Novo Nordisk Foundation Center for Genomic Mechanisms of Disease, Broad Institute of MIT and Harvard, Cambridge, MA USA; 4 The Charles Bronfman Institute for Personalized Medicine, Icahn School of Medicine at Mount Sinai, New York, NY USA

**Keywords:** Cardiovascular diseases, Follow-up studies, Obesity, Paediatric obesity, Preventive medicine, Public health, Type 2 diabetes, Weight gain

## Abstract

**Aims/hypothesis:**

Childhood overweight increases the risk of type 2 diabetes and cardiovascular disease in adulthood. However, the impact of childhood leanness on adult obesity and disease risk has been overlooked. We examined the independent and combined influences of child and adult body size on the risk of type 2 diabetes and cardiovascular disease.

**Methods:**

Data from the UK Biobank on 364,695 individuals of European ancestry and free of type 2 diabetes and cardiovascular disease were divided into nine categories based on their self-reported body size at age 10 and measured BMI in adulthood. After a median follow-up of 12.8 years, 33,460 individuals had developed type 2 diabetes and/or cardiovascular disease. We used Cox regression models to assess the associations of body size categories with disease incidence.

**Results:**

Individuals with low body size in childhood and high body size in adulthood had the highest risk of type 2 diabetes (HR 4.73; 95% CI 4.50, 4.99), compared to those with average body size in both childhood and adulthood. This was significantly higher than the risk in those with high body size in both childhood and adulthood (HR 4.05; 95% CI 3.84, 4.26). By contrast, cardiovascular disease risk was determined by adult body size, irrespective of childhood body size.

**Conclusions/interpretation:**

Low body size in childhood exacerbates the risk of type 2 diabetes associated with adult obesity but not the risk of cardiovascular disease. Thus, promoting healthy weight management from childhood to adulthood, among lean children, is crucial.

**Graphical Abstract:**

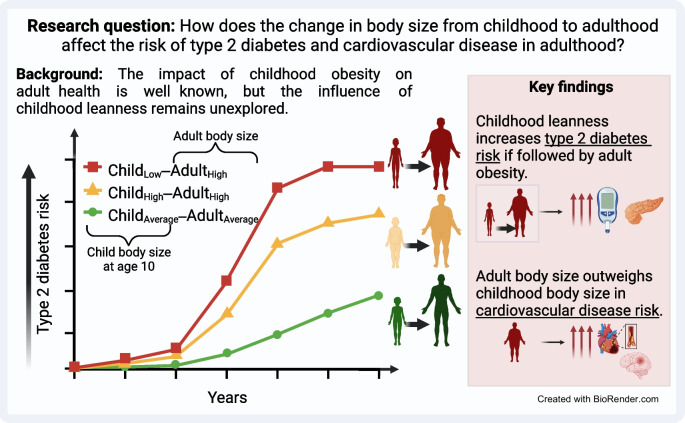

**Supplementary Information:**

The online version contains peer-reviewed but unedited supplementary material available at 10.1007/s00125-023-06058-4.


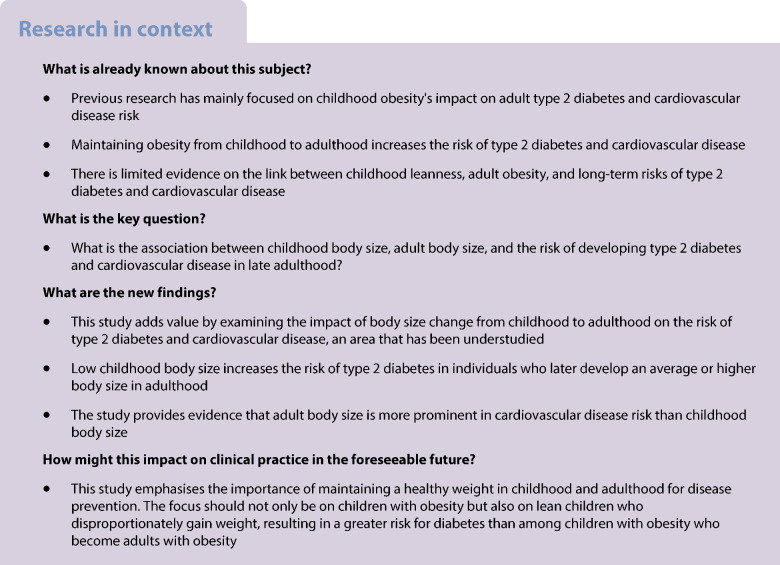


## Introduction

Childhood obesity has become a major public health concern in recent decades, with rates tripling over the past 30 years. By 2030, global estimates indicate that more than 100 million children (5–9 years) and more than 150 million adolescents (10–19 years) will be affected by obesity, representing 1 in 8 children and adolescents worldwide [1]. This is particularly concerning as children with obesity are at higher risk of carrying obesity through into adulthood, thus increasing the risk of type 2 diabetes and cardiovascular disease [1, 2].

While some studies suggest that the risk of type 2 diabetes in adulthood is reversible if children with overweight or obesity change to being of normal weight in adulthood [3, 4], others have shown that the risk of type 2 diabetes persists [5, 6]. Findings for cardiovascular risk factors [7], and for both metabolic and cardiovascular disease, are also inconsistent [5, 8, 9]. Additionally, studies that explore leanness in childhood and obesity in adulthood are inconclusive on the risk of type 2 diabetes and cardiovascular disease [10, 11]. Further, their follow-up period did not extend beyond middle age for cardiometabolic disease.

Large-scale studies with long-term follow-up are needed to elucidate the effects of childhood body size on type 2 diabetes and cardiovascular health in late adulthood. Thus, we examined the independent and combined effects of body size in childhood and adulthood on the risk of developing type 2 diabetes or cardiovascular disease later in life.

## Methods

### Study design and population

The present analyses are based on data from the UK Biobank, a prospective cohort of about 500,000 individuals aged 40 to 69 years at recruitment; participants were approximately 54% women and 46% men, predominantly of white ethnicity, and generally from higher socioeconomic backgrounds. Participants were invited to attend one of the 21 assessment centres in the UK between 2006 and 2010 to provide baseline information, physical measures, and biological samples according to standardised procedures [12]. Participants were followed up through linkage to healthcare records, including primary care, inpatient registries and death records, as of 1 February 2022. The register-based information was complemented with data obtained through online questionnaires at baseline and follow-up examinations [13].

**Fig. 1:**
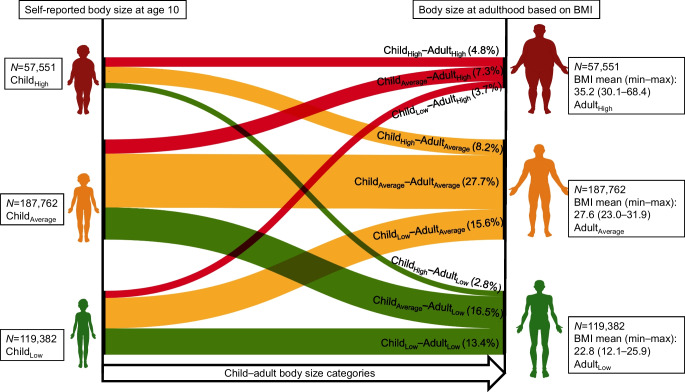
Definition and proportions for body size change from childhood to adulthood

We included unrelated individuals of European ancestry with data available from the enrolment examination on height and weight and on self-reported ‘comparative body size at age 10’. European ancestry was defined using the criteria provided by the Pan-UK Biobank team (https://pan.ukbb.broadinstitute.org; 2020). Related individuals were excluded using the Kinship-based INference for Genome-wide association studies (KING) algorithm [14]. Individuals with prevalent type 1 diabetes (*N*=1982), prior type 2 diabetes (*N*=6866) or cardiovascular disease (*N*=10,826) or both (*N*=4703) at baseline, and pregnant women (*N*=79) at baseline, were excluded (electronic supplementary material [ESM] Fig. 1).

The UK Biobank received ethical approval from the North West–Haydock Research Ethics Committee (11/NW/0382). The current study was conducted under the UK Biobank application 32683, and all participants provided informed consent at enrolment.

### Child-to-adult body size categories

Body weight and height in adulthood were measured by trained staff using standardised procedures at the enrolment assessment visit [15]. Measurements were taken without shoes and heavy clothing. Weight was measured using a Tanita BC-418MA scale (Amsterdam, Netherlands), and standing height was measured barefoot, using a Seca 202 device (Birmingham, UK). BMI was calculated by dividing body weight in kilograms by the square of height in metres.

Childhood body size was defined using data from the touchscreen questionnaire. Participants were asked the question: ‘When you were 10 years old, compared to average, would you describe yourself as: thinner, about average, or plumper?’. The answers were used to define three groups of childhood body size: ‘thinner’ (Child_Low_), ‘about average’ (Child_Average_) and ‘plumper’ (Child_High_), representing 32.7%, 51.5% and 15.8% of the study population, respectively. Of the included participants, 1.6% answered that they ‘Do not know’ (*N*=5869) or ‘Prefer not to answer’ (*N*=76) to that specific question and thus were excluded. The validity of this measure has been supported by previous studies investigating polygenic scores against measured BMI from British and Norwegian birth cohorts [16, 17].

We also tested the standard cut-offs for defining overweight and obesity according to the World Health Organization [18]. However, we could not find a corresponding adult low body size group large enough, including participants BMI≤18.5 (*N*=1892), which is 0.5% of the population. Further, most individuals fell into the overweight group (~66%, BMI≥25), equating to adult high body size. Thus, when categorising child-to-adult body size with these criteria, extreme groups were small and unbalanced in sample size and disease incidence. For this reason, we made adult body size as comparable as possible in sample size to child body size to allow properly balanced groups, and as previously done in earlier research [16].

Adult body size was estimated using BMI residuals from a model that included age, age squared, and self-reported sex as predictors (BMI  ~ age + age^2^ by sex). We created three adult body size groups of the same size as the corresponding childhood groups: Adult_Low_ (32.7%), Adult_Average_ (51.5%), and Adult_High_ (15.8%), with the corresponding adult BMI mean, minimum, and maximum values, as shown in Fig. 1, and with sex-stratified figures in ESM Fig. 2. Next, we created nine child–adult body size categories by combining the three child and three adult body size groups; proportions of these nine categories are reported in Fig. 1. Each category represents an individual's trajectory from a body size group in childhood to a body size group in adulthood (Fig. 1, ESM Fig. 2).

### Incident cardiovascular disease and type 2 diabetes

Fatal and non-fatal myocardial infarction or stroke were defined based on algorithmic combinations of linked data from hospital inpatient records (>94% of cases), death register records (~4–5%), and baseline questionnaire data on self-reported medical conditions, operations and medications (<0.5%). High-accuracy algorithms were developed by the UK Biobank outcome adjudication group [19, 20]. A cardiovascular composite endpoint was then created, using the first occurrence for either myocardial infarction or stroke to define the time-to-event for the cardiovascular endpoints.

The first occurrence of type 2 diabetes was ascertained from medical records including hospital inpatient records (87.8%), primary care records (9.8%) and death register records (0.3%), using ICD-9 codes (250) and ICD-10 codes (E11). This information was supplemented with baseline questionnaire data on self-reported medical conditions (2.1%) [13].

Time-to-event was defined from the date of the centre assessment in the UK Biobank to the date of the first occurrence of incident type 2 diabetes or cardiovascular diagnosis, date of death, being lost to follow-up, or end of follow-up. Follow-up time ended on 1 February 2022, as this was the latest date available for endpoint occurrence from the medical records. Time-to-event was also calculated separately for myocardial infarction and stroke.

### Covariates and potential confounders

Here, we selected age (years), sex (women/men), socioeconomic indicators (education; Townsend deprivation index) and assessment centre as potential confounders. Education was defined by the highest qualification achieved, converted into the International Standard Classification for Education (ISCED) (ESM Table 1). The Townsend deprivation index was calculated based on preceding national census output areas, with higher scores representing more socioeconomic deprivation [21].

We report baseline anthropometric measures and cardiometabolic risk markers across the nine body size categories. Anthropometric measures were assessed using BMI, WHR (calculated by dividing waist circumference [cm] by hip circumference [cm]) and body fat percentage (estimated by bioimpedance). Cardiometabolic risk markers, measured in serum samples at baseline, included glucose (mmol/l), HbA_1c_ (mmol/mol), total cholesterol (mmol/l), LDL-cholesterol (mmol/l), HDL-cholesterol (mmol/l), and triglycerides (mmol/l), all measured using Beckman Coulter AU5800 (UK) analyser, except for HbA_1c_, measured using Bio-Rad Variant II Turbo (Hertfordshire, UK) analyser. Systolic blood pressure (mmHg) was measured twice, a few moments apart, using an automated device (Omron, Hoofddorp, the Netherlands).

### Statistical analysis

Basic descriptive information over the nine child–adult body size categories was reported using means and standard deviations for continuous variables and *n* (%) for categorical variables. Differences in continuous variables were tested using one-way ANOVA, and in categorical variables using χ^2^ tests.

The Kaplan–Meier estimator was used to assess the association between the childhood or adulthood groups separately, and the nine child–adult body size categories and incidence of type 2 diabetes or cardiovascular disease. To quantify this association, time-to-event data was analysed using Cox proportional hazards regression, adjusted for potential confounders listed earlier to obtain HR and 95% CI. The reference category used was the Child_Average_–Adult_Average_ group. Sensitivity analyses were conducted to verify whether the risk of cardiovascular disease differed when considering the first occurrence of myocardial infarction or stroke separately. Furthermore, we included prevalent cases of cardiovascular disease and type 2 diabetes and recreated the nine child–adult body size categories to assess whether the results differ from incident disease analysis. Additionally, we performed a sensitivity analysis that included information on birthweight reported at baseline to see whether the main results were attenuated when adding birthweight as a covariate in the models. Furthermore, we made additional adjustments for various lifestyle factors (smoking status, dietary score, physical activity, sleep and sedentary time), family history of diabetes, and prevalent diseases (see ESM Methods) to assess whether any of these might be mediating or confounding our findings. Finally, we stratified by sex and defined age tertiles to assess the potential impact of body size changes from childhood to middle-aged or older adulthood. All analyses were stratified by sex and the results were compared.

In a post hoc analysis, we examined the impact of genetic factors on the observed associations between child-to-adult body size change and disease risk. Specifically, we considered genetic risk scores related to adult obesity to investigate whether differing genetic risks could explain the particularly high incidence of type 2 diabetes in individuals who were low-sized as children and high-sized as adults, compared with those who were high-sized during both childhood and adulthood (ESM Methods).

All statistical analyses were conducted using Stata 15.0 (StataCorp, College Station, TX, USA; www.stata.com).

## Results

Our study included data of 364,695 individuals (ESM Fig. 1) of whom 33,460 (9.2%) were diagnosed with incident type 2 diabetes (*N*=18,495) and/or cardiovascular disease (*N*=17,320) during a median follow-up time of 12.8 years (IQR 12.0–13.6 years). This corresponds to an incidence rate of 4.10 per 1000 person-years for type 2 diabetes (95% CI 4.04, 4.16) and 3.82 for cardiovascular disease (95% CI 3.76 to 3.87).

Many individuals remained in the same body size category from childhood to adulthood (45.9%), whereas 26.6% went from a lower body size group in childhood (Child_Average_ or Child_Low_) to a higher one in adulthood (Adult_Average_ or Adult_High_) and 27.5% went from a higher body size group in childhood (Child_High_ or Child_Average_) to a lower one in adulthood (Adult_Average_ or Adult_Low_) (Fig. 1, ESM Fig. 2). Notably, 19.3% of individuals had a low body size in childhood and an average or high body size in adulthood (Child_Low_−Adult_Average_ or Child_Low_−Adult_High_, Fig. 1). Baseline descriptive information across the nine child-to-adult categories is reported for all individuals in ESM Table 2 and for men and women separately in ESM Tables 3, 4. Those with a low childhood body size had somewhat smaller adult BMI but similar or higher (in women) WHR compared to those with a high childhood body size in each adult body size category (ESM Tables 2–4).

### Childhood or adulthood body size groups and disease incidence

Individuals with either high body size (Child_High_ HR 1.57 [95% CI 1.51, 1.63]) or low body size (Child_Low_ HR 1.23 [95% CI 1.19, 1.27]) in childhood have a higher incidence of type 2 diabetes compared to those with average body size (Child_Average_) (ESM Fig. 3). For cardiovascular disease, differences were more subtle, and only individuals with a high body size in childhood have a higher incidence (HR 1.12 [95% CI 1.07, 1.17]) compared to those with average body size (Child_Average_) (ESM Fig. 4). Distribution of adult BMI across these childhood groups by age tertiles and sex are reported in ESM Table 5.

Individuals with high body size (Adult_High_) in adulthood have a higher incidence (HR 3.47 [95% CI 3.37, 3.58]) and those with low body size have a lower incidence (Adult_Low_ HR 0.34 [95% CI 0.32, 0.36]) for type 2 diabetes compared to those with average body size (Adult_Average_) (ESM Fig. 3). Associations between adult body size and incidence of cardiovascular disease risk were consistent with those observed for type 2 diabetes, but much less pronounced (Adult_High_ HR 1.25 [95% CI 1.20, 1.30]); Adult_Low_ HR 0.83 [95% CI 0.80 to 0.86]) (ESM Fig. 4).

### Combined child–adult body size categories and disease incidence

**Fig. 2:**
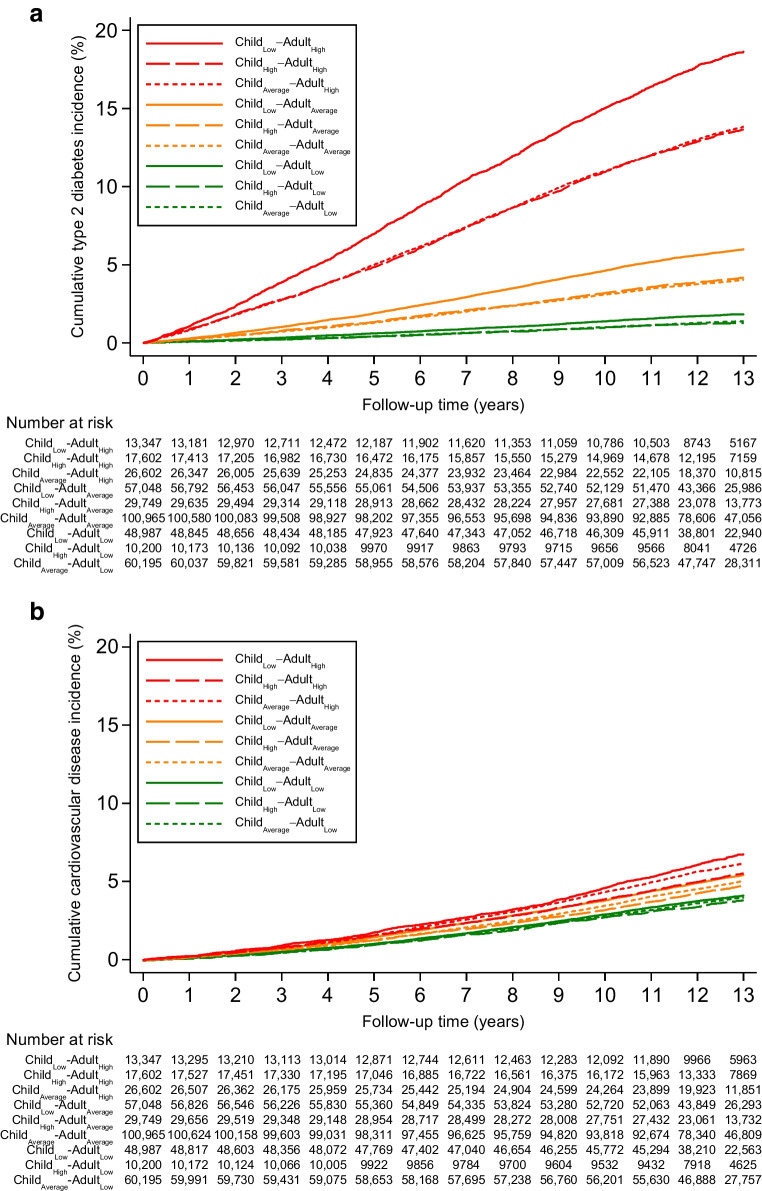
Type 2 diabetes and cardiovascular disease incidence by child–adult body size categories. Shown are the Kaplan–Meier estimates of incidence of disease across body size change categories and individuals at risk in each category

**Fig. 3:**
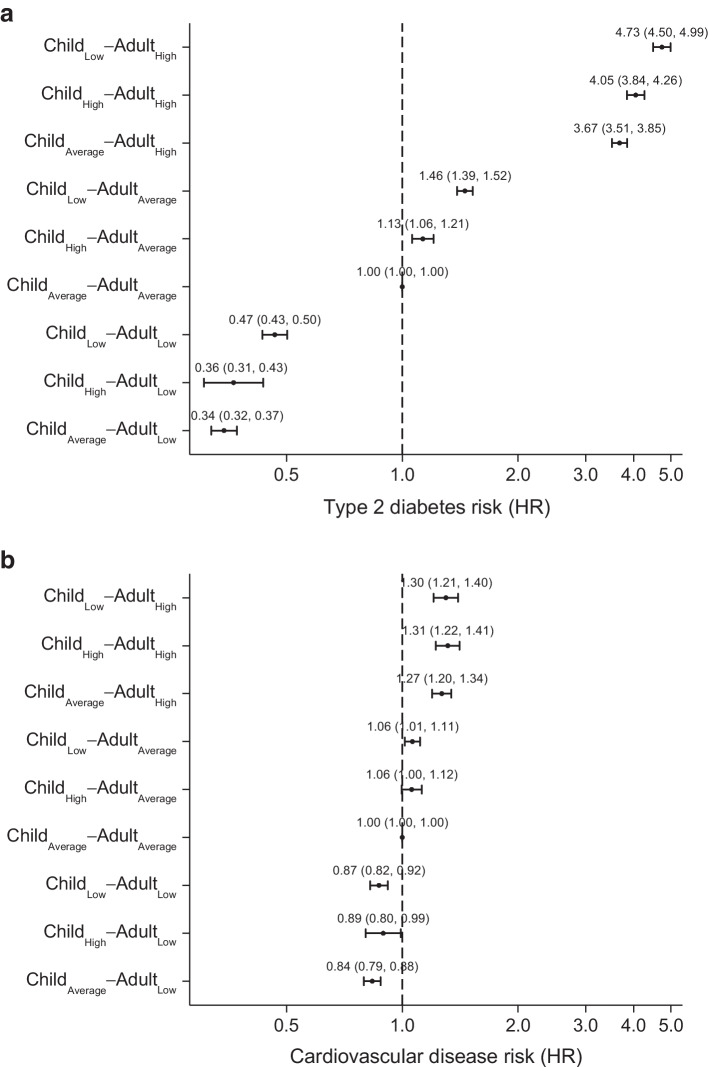
Risk of type 2 diabetes and cardiovascular disease by child–adult body size categories. Shown are adjusted HR for disease risk across body size change categories. In these comparisons, individuals in the Child_Average_–Adult_Average_ group served as the reference group. Cox regressions were adjusted for age, sex, educational attainment, Townsend deprivation index and assessment centre. Bars indicate 95% CI

Across the nine child–adult body size categories, it was the three categories with high adult body size that are associated with the highest incidence of type 2 diabetes, and the three categories with low adult body size that are associated with the lowest incidence (Figs 2, 3). However, within each adult body size category, individuals who had a low body size in childhood had a higher risk of incident type 2 diabetes than those who had a high or average body size in childhood (Figs 2, 3).

Specifically, individuals who had a low body size in childhood and a high body size in adulthood (Child_Low_–Adult_High_) had 4.73 (95% CI 4.50, 4.99) fold higher risk of type 2 diabetes compared to those with average body size in childhood and adulthood (Child_Average_–Adult_Average_). This was substantially higher than those who had a high or average body size in childhood and a high body size in adulthood (Child_High_–Adult_High_ HR 4.05 [95% CI 3.84, 4.26]; Child_High_–Adult_Average_ HR 3.67 [95% CI 3.51, 3.85]). The same pattern was observed in both women (Child_Low_–Adult_High_ HR 5.80 [95% CI 5.36, 6.28]) and men (HR 4.06 [95% CI 3.78, 4.35]) (ESM Fig. 5, 6). In addition, among individuals with average or low body size in adulthood, it was those with low body size in childhood who had a higher risk of type 2 diabetes.

For cardiovascular disease, individuals with high body size in adulthood have the highest incidence, whereas those with low adult body size have the lowest risk, although with effect size much smaller than those seen for type 2 diabetes (Fig. 3). Within each adult body size group, childhood body size did not further increase the risk of incident cardiovascular disease.

Sensitivity analyses for myocardial infarction (cases, *N*=10,871) and stroke (*N*=7061) separately showed similar results to those for both diseases combined (ESM Fig. 7, 8). Further, including prevalent cases of cardiovascular disease and type 2 diabetes did not change the findings compared with analysing only incident disease (ESM Fig. 9). Additionally, when we adjust for birthweight, the results remain unchanged (ESM Fig. 10). For this, we restricted our population to individuals with information on birthweight (*N*=212,416), recreated the nine child–adult categories, and reran the same Cox multivariable primary model as was done for type 2 diabetes (cases, *N*=9828) and cardiovascular disease (cases, *N*=8495) with and without adjustment for birthweight (ESM Fig. 10). Additional adjustments for various lifestyle factors, family history of diabetes, and prevalent diseases did not alter the main findings (ESM Methods and ESM Fig. 11). The sensitivity analysis, stratifying across age tertiles, revealed no significant differences compared with the primary analysis (ESM Fig. 12).

Stratified by sex, polygenic risk scores for adult obesity categorised individuals by high, medium and low genetic risk (ESM Fig. 13). Nevertheless, those with low body size in childhood and high body size as adults still showed the highest type 2 diabetes risk, consistent with the main results (ESM Fig. 13 and ESM Methods). Cardiovascular disease results also align with the main findings.

## Discussion

It has been well established that excess body weight in childhood and adulthood negatively impacts future cardiometabolic health compared to individuals with no excess body weight [22–24]. However, the influence of low body size in childhood is hardly ever studied. In this large retrospective and prospective study, we show that those with low body size in childhood, at any level of adult body size, have a higher risk of incident type 2 diabetes than those with high or average body size in childhood. The observed associations were consistent across women and men. As such, the highest risk of incident type 2 diabetes is incurred by individuals with low body size in childhood and high body size in adulthood. By contrast, adult body size determined the risk for cardiovascular disease, irrespective of childhood body size.

Research on the relationship between body size from childhood to adulthood and the risk of type 2 diabetes is limited [3, 10, 11, 25]. Previous longitudinal studies have mainly examined the impact of high body size during childhood transitioning into adulthood. However, there have been only a few limited-in-scope analyses on the role of low body size during childhood concerning the future risk of adult cardiometabolic disease [10, 11].

A study of over 80,000 women in the E3N cohort found that those with a lean body shape at age 8 and a significant increase in body size from childhood to adulthood had a higher risk of type 2 diabetes compared with women who maintained a stable body shape [10]. Similar findings were observed in the Nurses' Health Study (*N*=69,598 women) and the Health Professionals Follow-up Study (*N*=30,910 men) [11]. For women, those who were lean in childhood but gained weight in adulthood had the highest risk of type 2 diabetes. For men, this group was the second highest in risk, closely following those who were heavy in childhood and gained more weight in adulthood. In this study, the risk of type 2 diabetes attributed to weight gain and heavy body size was relatively low in men compared with women, and body shape was measured by means of pictorial body diagrams, which may not have provided a comparable assessment of body size between men and women. Nonetheless, these observations are generally consistent with our findings that individuals with low body size in childhood and high body size in adulthood have the highest risk of type 2 diabetes in adulthood.

Several studies have examined the impact of childhood obesity, but not low body size in childhood, on the risk of type 2 diabetes. These studies consistently found that individuals with persistent obesity from childhood to adulthood have a substantially increased risk of type 2 diabetes. However, those who had obesity in childhood but not in adulthood had either no increased risk or a modestly increased risk compared with those who never had obesity [3, 4, 7, 9, 26–29]. Two studies, based on the US National Longitudinal Study of Adolescent Health (*N*=10,481) [26] and the Young Finns Study (*N*=2631) [7], reported an increased risk of adult type 2 diabetes in individuals with persistent child-to-adult obesity, while those with childhood obesity but not adult obesity had the same risk as those who never had obesity. Similarly, a Danish study (*N*=62,565 men) showed that individuals with persistent child-to-adult overweight had a substantially increased risk of type 2 diabetes, whereas those who had overweight at age 7 but not at a later age had a modestly increased risk compared with those who had never had overweight [4]. A recent study found that childhood obesity has a protective effect on insulin resistance when conditioned on adult BMI [30]. Our findings show that high childhood body size is only ‘protective’ when compared to children with a low body size in childhood who reach a high body size in adulthood. Nevertheless, children with high body size and persistent adult obesity have a higher type 2 diabetes risk than the average population.

The evidence of an added impact of childhood body size is less clear for cardiovascular disease than for type 2 diabetes. Studies on BMI change and cardiovascular disease risk showed that a greater increase in BMI is associated with increased cardiovascular risk [8, 24, 31, 32]. Consistent with our observations, some studies found that childhood body size did not increase the risk beyond the effect of obesity in adulthood [11, 33, 34], and also that child-to-adult obesity persistence increases the risk, whereas remission from childhood obesity into normal body weight in adulthood decreases cardiovascular risk [5, 7, 11, 33–35]. However, a study based on three British cohorts (*N*=11,447) found that, compared with those who were never overweight, individuals who transitioned from normal weight in childhood and non-obese in adolescence to obesity in adulthood had a higher risk of coronary heart disease than those who were obese in both childhood and adulthood [36]. The latter study did not consider a childhood lean weight group as a separate category, as they were combined in the normal weight group instead.

Previous studies indicated that birthweight is a risk factor for type 2 diabetes and cardiovascular disease [37–39]. Thus, we decided to explore how this may affect our main findings. Sensitivity analysis controlling for birthweight supports that child-to-adult body size is an independent risk factor for cardiometabolic disease.

Our original contribution that children with a low body size in childhood have an increased risk of developing type 2 diabetes if they develop average or higher body size in adulthood, may be due to differences in the number of adipocytes available for storing fat [40]. The development of metabolic dysfunction during weight gain is closely connected to adipocyte hypertrophy (i.e. larger adipocytes), whereas adipocyte hyperplasia (i.e. more adipocytes) may buffer against metabolic dysfunction [41]. The capacity for hyperplasia is greater in children than in adults [42], and thus heavier children may develop a larger number of adipocytes than those who are lean [43, 44]. As a result, individuals with a low body size in childhood may be particularly susceptible to metabolic dysfunction upon weight gain, by having a small number of adipocytes available for storing fat. In support of this hypothesis, we observed a similar or higher WHR (for women only) in those with a low childhood body size compared to those with a high childhood body size in each adult body size category, despite having a somewhat smaller BMI, which suggests an impaired capacity to store subcutaneous fat.

Our study is noteworthy for its comprehensive approach, including long-term childhood-to-adulthood follow-up, a large sample size, and objectively and accurately defined incident disease endpoints. Nevertheless, there are some limitations to consider. Childhood body size was self-reported, which may introduce recall bias. However, the self-reported childhood body size variable was found to be somewhat consistent with objectively measured childhood BMI [16, 17]. Furthermore, the way we categorised childhood and adulthood body size may lack practical meaning and comparability with other studies. We also did not have repeated body size measures, such as height, weight or BMI, in childhood or adulthood, such that corresponding weight fluctuations could not be captured. We had information on socioeconomic status in adulthood but not childhood. As such, we could not determine if changes in body size were linked to changes in socioeconomic status, which can affect weight differently in childhood and adulthood [45]. The absence of data on childhood diet and physical activity, and their changes into adulthood, may introduce residual confounding. We note that our analyses were performed in individuals of European ancestry only; therefore, our findings may not be generalisable to other populations.

### Conclusion

Our findings show that low body size in childhood is a risk factor for type 2 diabetes among those who later develop an average or higher body size in adulthood. By contrast, adult body size was the leading risk factor for cardiovascular disease, irrespective of childhood body size. Our results highlight the need to promote healthy weight management from childhood to adulthood and reveal that lean children are also vulnerable under certain circumstances.

### Supplementary Information

Below is the link to the electronic supplementary material.

Supplementary file1 (PDF 3.52 MB)

## Data Availability

The UK Biobank data are available to any researcher worldwide upon application to the UK Biobank (www.ukbiobank.ac.uk).

## References

[1] Lobstein T, Brinsden H, Neveux M (2022) World Obesity Atlas 2022. Available from https://s3-eu-west-1.amazonaws.com/wof-files/World_Obesity_Atlas_2022.pdf

[2] Simmonds M, Llewellyn A, Owen CG, Woolacott N (2016). Predicting adult obesity from childhood obesity: a systematic review and meta-analysis. Obes Rev.

[3] Yeung EH, Zhang C, Louis GM, Willett WC, Hu FB (2010). Childhood size and life course weight characteristics in association with the risk of incident type 2 diabetes. Diabetes Care.

[4] Bjerregaard LG, Jensen BW, Angquist L, Osler M, Sorensen TIA, Baker JL (2018). Change in overweight from childhood to early adulthood and risk of type 2 diabetes. N Engl J Med.

[5] Juonala M, Magnussen CG, Berenson GS (2011). Childhood adiposity, adult adiposity, and cardiovascular risk factors. N Engl J Med.

[6] Liang Y, Hou D, Zhao X (2015). Childhood obesity affects adult metabolic syndrome and diabetes. Endocrine.

[7] Buscot MJ, Thomson RJ, Juonala M (2018). Distinct child-to-adult body mass index trajectories are associated with different levels of adult cardiometabolic risk. Eur Heart J.

[8] Ohlsson C, Bygdell M, Sonden A, Rosengren A, Kindblom JM (2016). Association between excessive BMI increase during puberty and risk of cardiovascular mortality in adult men: a population-based cohort study. Lancet Diabetes Endocrinol.

[9] Sun J, Xi B, Yang L, Zhao M, Juonala M, Magnussen CG (2021). Weight change from childhood to adulthood and cardiovascular risk factors and outcomes in adulthood: a systematic review of the literature. Obes Rev.

[10] Fagherazzi G, Vilier A, Affret A, Balkau B, Bonnet F, Clavel-Chapelon F (2015). The association of body shape trajectories over the life course with type 2 diabetes risk in adulthood: a group-based modeling approach. Ann Epidemiol.

[11] Zheng Y, Song M, Manson JE, Giovannucci EL, Hu FB (2017). Group-based trajectory of body shape from ages 5 to 55 years and cardiometabolic disease risk in 2 US cohorts. Am J Epidemiol.

[12] Hewitt J, Walters M, Padmanabhan S, Dawson J (2016). Cohort profile of the UK Biobank: diagnosis and characteristics of cerebrovascular disease. BMJ Open.

[13] UK Biobank (2019) First occurrence of health outcomes defined by 3-character ICD10 code. UK Biobank, Stockport

[14] Manichaikul A, Mychaleckyj JC, Rich SS, Daly K, Sale M, Chen WM (2010). Robust relationship inference in genome-wide association studies. Bioinformatics.

[15] Collins R (2007) UK Biobank: protocol for a large-scale prospective epidemiological resource. Available from https://www.ukbiobank.ac.uk/media/gnkeyh2q/study-rationale.pdf

[16] Richardson TG, Sanderson E, Elsworth B, Tilling K, Davey Smith G (2020). Use of genetic variation to separate the effects of early and later life adiposity on disease risk: Mendelian randomisation study. BMJ.

[17] Brandkvist M, Bjorngaard JH, Odegard RA (2021). Separating the genetics of childhood and adult obesity: a validation study of genetic scores for body mass index in adolescence and adulthood in the HUNT Study. Hum Mol Genet.

[18] World Health Organization (2022) WHO European regional obesity report 2022. WHO Regional Office for Europe, Copenhagen

[19] Rubbo B, Fitzpatrick NK, Denaxas S (2015). Use of electronic health records to ascertain, validate and phenotype acute myocardial infarction: a systematic review and recommendations. Int J Cardiol.

[20] Rannikmäe K, Ngoh K, Bush K (2020). Accuracy of identifying incident stroke cases from linked health care data in UK Biobank. Neurology.

[21] Samarasundera E, Martin D, Saxena S, Majeed A (2010). Socio-demographic data sources for monitoring locality health profiles and geographical planning of primary health care in the UK. Prim Health Care Res Dev.

[22] Twig G, Yaniv G, Levine H (2016). Body-mass index in 2.3 million adolescents and cardiovascular death in adulthood. N Engl J Med.

[23] Tirosh A, Shai I, Afek A (2011). Adolescent BMI trajectory and risk of diabetes versus coronary disease. N Engl J Med.

[24] Jacobs DR, Woo JG, Sinaiko AR (2022). Childhood cardiovascular risk factors and adult cardiovascular events. N Engl J Med.

[25] Golozar A, Khademi H, Kamangar F (2011). Diabetes mellitus and its correlates in an Iranian adult population. PLoS One.

[26] The NS, Richardson AS, Gordon-Larsen P (2013). Timing and duration of obesity in relation to diabetes: findings from an ethnically diverse, nationally representative sample. Diabetes Care.

[27] Bjerregaard LG, Wasenius N, Nedelec R (2020). Possible modifiers of the association between change in weight status from child through adult ages and later risk of type 2 diabetes. Diabetes Care.

[28] Abraham S, Collins G, Nordsieck M (2016). Relationship of childhood weight status to morbidity in adults. Int J Epidemiol.

[29] Zhang T, Xu J, Li S (2019). Trajectories of childhood BMI and adult diabetes: the Bogalusa Heart Study. Diabetologia.

[30] Hawkes G, Beaumont RN, Tyrrell J et al (2023) Genetic evidence that high BMI in childhood has a protective effect on intermediate diabetes traits, including measures of insulin sensitivity and secretion, after accounting for BMI in adulthood. Diabetologia 66(8):1472–1480. 10.1007/s00125-023-05923-6 10.1007/s00125-023-05923-6 PMC10317883 37280435

[31] Morrison JA, Glueck CJ, Woo JG, Wang P (2012). Risk factors for cardiovascular disease and type 2 diabetes retained from childhood to adulthood predict adult outcomes: the Princeton LRC Follow-up Study. Int J Pediatr Endocrinol.

[32] Baker JL, Olsen LW, Sorensen TI (2007). Childhood body-mass index and the risk of coronary heart disease in adulthood. N Engl J Med.

[33] Lloyd LJ, Langley-Evans SC, McMullen S (2010). Childhood obesity and adult cardiovascular disease risk: a systematic review. Int J Obes (Lond).

[34] Park MH, Falconer C, Viner RM, Kinra S (2012). The impact of childhood obesity on morbidity and mortality in adulthood: a systematic review. Obes Rev.

[35] Ohlsson C, Bygdell M, Sonden A, Jern C, Rosengren A, Kindblom JM (2017). BMI increase through puberty and adolescence is associated with risk of adult stroke. Neurology.

[36] Park MH, Sovio U, Viner RM, Hardy RJ, Kinra S (2013). Overweight in childhood, adolescence and adulthood and cardiovascular risk in later life: pooled analysis of three British birth cohorts. PLoS One.

[37] Knop MR, Geng TT, Gorny AW (2018). Birth weight and risk of type 2 diabetes mellitus, cardiovascular disease, and hypertension in adults: a meta-analysis of 7 646 267 participants from 135 studies. J Am Heart Assoc.

[38] Hansen AL, Thomsen RW, Brons C et al (2023) Birthweight is associated with clinical characteristics in people with recently diagnosed type 2 diabetes. Diabetologia 66(9):1680–1692. 10.1007/s00125-023-05936-1 10.1007/s00125-023-05936-1 PMC10390374 37303007

[39] Wibaek R, Andersen GS, Linneberg A et al (2023) Low birthweight is associated with a higher incidence of type 2 diabetes over two decades independent of adult BMI and genetic predisposition. Diabetologia 66(9):1669–1679. 10.1007/s00125-023-05937-0 10.1007/s00125-023-05937-0 PMC10390608 37303008

[40] Carobbio S, Pellegrinelli V, Vidal-Puig A (2017). Adipose tissue function and expandability as determinants of lipotoxicity and the metabolic syndrome. Adv Exp Med Biol.

[41] Pellegrinelli V, Carobbio S, Vidal-Puig A (2016). Adipose tissue plasticity: how fat depots respond differently to pathophysiological cues. Diabetologia.

[42] Landgraf K, Rockstroh D, Wagner IV (2015). Evidence of early alterations in adipose tissue biology and function and its association with obesity-related inflammation and insulin resistance in children. Diabetes.

[43] Rodriguez-Cuenca S, Vidal-Puig A (2021). Insulin and the last gasp of failing adipocytes. Nat Metab.

[44] Li Q, Hagberg CE, Silva Cascales H (2021). Obesity and hyperinsulinemia drive adipocytes to activate a cell cycle program and senesce. Nat Med.

[45] Bann D, Johnson W, Li L, Kuh D, Hardy R (2017). Socioeconomic inequalities in body mass index across adulthood: coordinated analyses of individual participant data from three British birth cohort studies initiated in 1946, 1958 and 1970. PLoS Med.

