# Supplementary material for: Child-to-adult body size change and risk of type 2 diabetes and cardiovascular disease
Source: Diabetologia. 2023 Dec 12;67(5):864–73. doi: 10.1007/s00125-023-06058-4 (PMC10954919; doi:10.1007/s00125-023-06058-4)

Electronic supplementary material (ESM)

Title: Child-to-adult body size change and risk of type 2 diabetes and cardiovascular disease

Authors: Carrasquilla et al

ESM Methods

Lifestyle factors, family history, and prevalent disease definitions

We defined healthy diet scores based on shared features from the World Health Organization recommendations [1] and traditional healthy Western and Oriental diets [2]. Such diets predominantly emphasize an increased intake of vegetables, fresh fruits, whole grains, and healthy fats while recommending reduced consumption of fatty and processed meats and salt. To gather data, we used a touchscreen questionnaire, which captured participants' reported weekly intake of various food and drink items as part of a food frequency questionnaire. The healthy diet score was calculated by summing daily servings of cooked and fresh vegetables and fresh fruits. It further considered factors such as consumption of whole grain bread (1 for yes, 0 for no), bran/oatmeal cereal (1 for yes, 0 for no), one healthy fish serving per week (1 for yes, 0 for no), ≤ 1 processed meat serving per week (0 for yes, 1 for no), ≤ 1 red meat serving per week (0 for yes, 1 for no), and the addition of extra salt to food (0 for yes, 1 for no).

Sedentary time was assessed considering data from three activities: driving, computer use, and television watching time. If an individual reported any of these activities, that value was taken into account. When two or three activities were reported, the mean value was used. This provided a single measure of sedentary behaviour for each individual.

Physical activity time (minutes per day) was defined by combining three distinct types of physical activities: walking, moderate physical activity, and vigorous physical activity. To consider the diverse intensities and contributions of these activities, we applied distinct weightings: 1 for walking (lighter intensity), 2 for moderate activity (moderate intensity), and 3 for vigorous activity (higher intensity). To ensure that our measure of physical activity remained robust, we accommodated missing data by implementing an imputation strategy. Specifically, we calculated the mean value for situations with available data and replaced missing values with this calculated mean.

Smoking status was defined into three categories: current smokers, former smokers, or individuals who have never smoked tobacco products.

Family history of diabetes encompassed the presence or absence of a medical history of diabetes mellitus within an individual's immediate family, including their mother, father, or siblings, where one or more of these relatives had received a diagnosis of diabetes mellitus.

Prevalent cases were defined as medical conditions that have been previously diagnosed by a doctor, including diseases such as cancer and severe respiratory illnesses (i.e., chronic obstructive pulmonary disease).

Genome-wide association analysis and polygenic scores for adult body mass index

We conducted a genome-wide association study (GWAS) to elucidate the genetic determinants of adult body mass index (BMI) utilizing the REGENIE software [3] that accounts for relatedness, including the following covariates: age, sex, genotype chip, assessment centre, and the first 15 genetic principal components.

A total of 418,133 European individuals were enrolled in this analysis. Details about the genotyping and single nucleotide polymorphism (SNP) imputation procedure have been previously outlined [4]. This study focused on 776 variants that reached genome-wide significance and were independent (p<5x10-9, R2<0.1 and ±500 kilobases window) to be considered further for constructing polygenic risk scores.

We obtained a weighted polygenic risk score (PRS) using recently validated polygenic score methods in the UK Biobank [5]. Each SNP was categorized as having 0, 1, or 2 risk-increasing alleles based on their genetic makeup. These values were then multiplied by their respective effect sizes. These scores were then split into tertiles to define individuals with low, medium, and high genetic risk for adult obesity. We then rerun Cox models for the groups of interest.

Supplementary references

1. World Health Organization. "Action framework for developing and implementing public food procurement and service policies for a healthy diet." (2021).
2. Cena, Hellas, and Philip C. Calder. "Defining a healthy diet: evidence for the role of contemporary dietary patterns in health and disease." Nutrients 12.2 (2020): 334.
3. Mbatchou, Joelle, et al. "Computationally efficient whole-genome regression for quantitative and binary traits." Nature genetics 53.7 (2021): 1097-1103.
4. Bycroft, Clare, et al. "The UK Biobank resource with deep phenotyping and genomic data." Nature 562.7726 (2018): 203-209.
5. Collister, Jennifer A., Xiaonan Liu, and Lei Clifton. "Calculating polygenic risk scores (PRS) in UK Biobank: a practical guide for epidemiologists." Frontiers in genetics 13 (2022): 818574

ESM Table 1. Education qualifications based on the International Standard Classification										
	ChildLow	ChildAverage	ChildHigh	ChildLow	ChildAverage	ChildHigh	ChildLow	ChildAverage	ChildHigh	Total
**Characteristic**	AdultLow			AdultAverage			AdultHigh			
**College or University degree**	19,974 (41·1)	23,573 (39·5)	4,351 (43·0)	15,831 (28·0)	31,221 (31·2)	9,946 (33·7)	2,478 (18·8)	6,454 (24·5)	4,959 (28·4)	11,8787 (32·9)
**A levels/AS levels or equivalent**	6,096 (12·5)	7,244 (12·1)	1,199 (11·8)	6,204 (11·0)	11,618 (11·6)	3,366 (11·4)	1,192 (9·1)	3,019 (11·5)	2,072 (11·9)	42,010 (11·6)
**O levels/GCSEs or equivalent**	9,686 (19·9)	12,445 (20·8)	1,923 (19·0)	12,503 (22·1)	23,621 (23·6)	6,369 (21·6)	2,842 (21·6)	6,391 (24·3)	4,047 (23·2)	79,827 (22·1)
**CSEs or equivalent**	2,205 (4·5)	2,710 (4·5)	466 (4·6)	3,328 (5·9)	5,548 (5·5)	1,633 (5·5)	1,041 (7·9)	1,755 (6·7)	1,201 (6·9)	19,887 (5·5)
**NVQ or HND or HNC or equivalent**	2,732 (5·6)	3,234 (5·4)	485 (4·8)	4,223 (7·5)	6,788 (6·8)	1,878 (6·4)	1,100 (8·4)	1,949 (7·4)	1,217 (7·0)	23,606 (6·5)
**Other professional qualifications eg: nursing, teaching**	2,154 (4·4)	2,849 (4·8)	489 (4·8)	3,037 (5·4)	5,420 (5·4)	1,515 (5·1)	714 (5·4)	1,557 (5·9)	969 (5·6)	18,704 (5·2)
**Other**	5,781 (11·9)	7,679 (12·9)	1,215 (12·0)	11,369 (20·1)	15,844 (15·8)	4,811 (16·3)	3,796 (28·8)	5,202 (19·8)	2,987 (17·1)	58,684 (16·2)
**Total**	48,628 (13·5)	59,734 (16·5)	10,128 (2·8)	56,495 (15·6)	100,060 (27·7)	29,518 (8·2)	13,163 (3·6)	26,327 (7·3)	17,452 (4·8)	361,505 (100)

Results are reported as number of individuals (percentage)

ESM Table 2. Baseline characteristics across child to adult body size categories.

Characteristic	ChildLow	ChildAverage	ChildHigh	ChildLow	ChildAverage	ChildHigh	ChildLow	ChildAverage	ChildHigh	Total
	AdultLow			AdultAverage			AdultHigh			
No· of participants at baseline (%)	48,987	60,195	10,200	57,048	100,965	29,749	13,347	26,602	17,602	364,695
Age (years)	55·9 (8·0)	56·6 (7·9)	56·0 (7·6)	57·1 (8·0)	57·0 (8·0)	56·1 (7·9)	57·1 (7·9)	56·7 (7·8)	55·3 (7·8)	56·6 (8·0)
No· of female (%)	23,577 (48·1)	32,729 (54·4)	6,208 (60·9)	30,887 (54·1)	54,895 (54·4)	17,981 (60·4)	8,050 (60·3)	16,139 (60·7)	11,404 (64·8)	201,870 (55·4)
Educational attainment (university)	19,974 (41·1)	23,573 (39·5)	4,351 (43·0)	15,831 (28·0)	31,221 (31·2)	9,946 (33·7)	2,478 (18·8)	6,454 (24·5)	4,959 (28·4)	11,8787 (32·9)
Townsend deprivation index	-1·7 (2·9)	-1·7 (2·9)	-1·7 (3·0)	-1·6 (2·9)	-1·8 (2·8)	-1·6 (2·9)	-0·7 (3·2)	-1·2 (3·0)	-1·0 (3·1)	-1·6 (2·9)
Height (cm)	171·0 (9·3)	169·1 (8·8)	168·6 (9·1)	169·1 (9·5)	168·5 (9·0)	167·9 (9·2)	167·0 (9·6)	166·9 (9·2)	166·8 (9·2)	168·7 (9·2)
Body mass index (kg/m^2)	22·7 (1·9)	22·9 (1·7)	22·9 (1·7)	27·5 (1·9)	27·6 (2·0)	27·9 (2·0)	34·8 (3·4)	34·8 (3·4)	36·0 (4·4)	27·2 (4·7)
Waist-to-hip ratio	0·84 (0·1)	0·83 (0·1)	0·82 (0·1)	0·88 (0·1)	0·87 (0·1)	0·87 (0·1)	0·92 (0·1)	0·91 (0·1)	0·91 (0·1)	0·87 (0·1)
Body fat percentage (%)	25·1 (6·7)	25·1 (6·5)	26·1 (6·4)	32·7 (6·8)	32·1 (6·8)	33·3 (6·7)	40·4 (7·1)	40·0 (7·2)	41·2 (7·1)	31·3 (8·5)
Glucose levels (mmol/L)	4·9 (0·8)	4·9 (0·7)	4·9 (0·7)	5·1 (0·9)	5·0 (0·8)	5·0 (0·9)	5·3 (1·3)	5·3 (1·3)	5·2 (1·3)	5·0 (0·9)
Glycated haemoglobin (HbA1c) (mmol/mol)	34·5 (4·2)	34·4 (3·9)	34·2 (3·8)	35·6 (5·1)	35·2 (4·7)	35·0 (5·0)	37·9 (7·2)	37·2 (6·8)	37·0 (6·9)	35·3 (5·1)
Glycated haemoglobin (HbA1c) (%)	5·3 (0·4)	5·3 (0·4)	5·3 (0·3)	5·4 (0·5)	5·4 (0·4)	5·4 (0·5)	5·6 (0·7)	5·6 (0·6)	5·5 (0·6)	5·4 (0·5)
Systolic blood pressure (mm/Hg)	136·5 (19·6)	135·9 (19·8)	133·8 (19·8)	142·2 (19·3)	141·2 (19·4)	139·2 (19·3)	145·1 (18·8)	144·6 (18·8)	143·2 (18·9)	140·0 (19·6)
Cholesterol levels (mmol/L)	5·7 (1·1)	5·7 (1·0)	5·7 (1·0)	5·9 (1·1)	5·9 (1·1)	5·8 (1·1)	5·7 (1·2)	5·7 (1·1)	5·7 (1·1)	5·8 (1·1)
LDL cholesterol (mmol/L)	3·5 (0·8)	3·5 (0·8)	3·5 (0·8)	3·7 (0·9)	3·7 (0·9)	3·6 (0·9)	3·6 (0·9)	3·7 (0·9)	3·6 (0·9)	3·6 (0·8)
HDL cholesterol (mmol/L)	1·6 (0·4)	1·6 (0·4)	1·7 (0·4)	1·4 (0·3)	1·4 (0·4)	1·5 (0·4)	1·3 (0·3)	1·3 (0·3)	1·3 (0·3)	1·5 (0·4)
Triglycerides (mmol/L)	1·5 (0·8)	1·4 (0·7)	1·3 (0·7)	1·9 (1·1)	1·8 (1·0)	1·7 (1·0)	2·2 (1·2)	2·1 (1·1)	2·1 (1·1)	1·7 (1·0)
No· of incident incident type 2 diabetes (%)	868 (1·8)	795 (1·3)	129 (1·3)	3,294 (5·8)	3,947 (3·9)	1,201 (4·0)	2,403 (18·0)	3,544 (13·3)	2,314 (13·1)	18,495 (5·1)
No· of incident incident cardiovascular disease (%)	1,977 (4·0)	2,340 (3·9)	373 (3·7)	3,004 (5·3)	4,910 (4·9)	1,359 (4·6)	858 (6·4)	1,566 (5·9)	933 (5·3)	17320 (4·7)

Results are reported as number of individuals (percentage) for categorical variables and mean coefficients and standard deviations for continuous variables.

ESM Table 3. Baseline characteristics for women across child to adult body size categories.

Characteristic in female inviduals	ChildLow	ChildAverage	ChildHigh	ChildLow	ChildAverage	ChildHigh	ChildLow	ChildAverage	ChildHigh	Total
	AdultLow			AdultAverage			AdultHigh			
**No. of participants at baseline**	23,577	32,729	6,208	30,887	54,895	17,981	8,050	16,139	11,404	201,870
**Age (years)**	55·6 (8·0)	56·4 (7·8)	56·1 (7·6)	57·1 (7·9)	57·0 (7·9)	56·1 (7·8)	57·2 (7·8)	56·7 (7·8)	55·3 (7·8)	56·5 (7·9)
**Educational attainment (university degree) (%)**	8,973 (38·4)	12,583 (38·7)	2,472 (40·1)	7,970 (26·1)	16,563 (30·4)	5,704 (32·0)	1,440 (18·2)	3,918 (24·5)	3,155 (27·9)	62,778 (31·4)
**Townsend deprivation index**	-1·8 (2·8)	-1·8 (2·8)	-1·7 (2·9)	-1·6 (2·9)	-1·8 (2·8)	-1·6 (2·9)	-0·7 (3·2)	-1·2 (3·0)	-1·0 (3·1)	-1·6 (2·9)
**Height (cm)**	164·3 (6·4)	163·4 (6·0)	163·3 (6·2)	162·9 (6·5)	162·5 (6·0)	162·4 (6·1)	161·4 (6·5)	161·6 (6·0)	161·9 (6·2)	162·8 (6·2)
**Body mass index (kg/m^2)**	21·8 (1·6)	22·1 (1·5)	22·2 (1·5)	26·8 (2·0)	26·9 (2·0)	27·3 (2·0)	34·8 (3·6)	34·9 (3·6)	36·1 (4·6)	26·9 (5·0)
**Waist-to-hip ratio**	0·78 (0·1)	0·77 (0·1)	0·78 (0·1)	0·83 (0·1)	0·82 (0·1)	0·82 (0·1)	0·87 (0·1)	0·86 (0·1)	0·86 (0·1)	0·81 (0·1)
**Body fat percentage (%)**	30·0 (4·9)	29·3 (4·7)	29·6 (4·8)	38·0 (3·9)	37·2 (4·1)	37·7 (4·1)	45·4 (3·6)	44·9 (3·6)	45·6 (4·0)	36·4 (6·8)
**Glucose levels (mmol/L)**	4·9 (0·7)	4·9 (0·7)	4·9 (0·7)	5·0 (0·8)	5·0 (0·8)	5·0 (0·8)	5·3 (1·2)	5·2 (1·1)	5·1 (1·1)	5·0 (0·8)
**Glycated haemoglobin (HbA1c) (mmol/mol)**	34·5 (3·8)	34·4 (3·6)	34·2 (3·5)	35·5 (4·7)	35·0 (4·2)	34·8 (4·5)	37·5 (6·4)	36·9 (5·9)	36·6 (6·0)	35·2 (4·6)
**Glycated haemoglobin (HbA1c) (%)**	5·3 (0·3)	5·3 (0·3)	5·3 (0·3)	5·4 (0·4)	5·4 (0·4)	5·3 (0·4)	5·6 (0·6)	5·5 (0·5)	5·5 (0·5)	5·4 (0·4)
**Systolic blood pressure (mm/Hg)**	133·2 (20·3)	132·8 (20·2)	131·1 (20·1)	139·6 (20·1)	138·3 (20·1)	136·4 (19·5)	143·4 (19·1)	142·7 (19·2)	141·3 (19·0)	137·3 (20·2)
**Cholesterol levels (mmol/L)**	5·8 (1·1)	5·9 (1·1)	5·8 (1·0)	6·0 (1·1)	6·0 (1·1)	5·9 (1·1)	5·9 (1·1)	5·9 (1·1)	5·9 (1·1)	5·9 (1·1)
**LDL cholesterol (mmol/L)**	3·5 (0·8)	3·5 (0·8)	3·5 (0·8)	3·8 (0·9)	3·7 (0·9)	3·7 (0·9)	3·7 (0·9)	3·7 (0·9)	3·7 (0·9)	3·7 (0·9)
**HDL cholesterol (mmol/L)**	1·7 (0·4)	1·8 (0·4)	1·8 (0·4)	1·6 (0·3)	1·6 (0·4)	1·6 (0·4)	1·4 (0·3)	1·4 (0·3)	1·4 (0·3)	1·6 (0·4)
**Triglycerides (mmol/L)**	1·3 (0·7)	1·2 (0·6)	1·2 (0·6)	1·7 (0·9)	1·6 (0·8)	1·5 (0·8)	2·0 (1·0)	1·9 (0·9)	1·9 (1·0)	1·5 (0·8)
**No. of incident incident type 2 diabetes (%)**	263 (1·1)	260 (0·8)	55 (0·9)	1,311 (4·2)	1,368 (2·5)	473 (2·6)	1,224 (15·2)	1,689 (10·5)	1,212 (10·6)	7,855 (3·9)
**No. of incident incident cardiovascular disease (%)**	572 (2·4)	821 (2·5)	162 (2·6)	1,171 (3·8)	1,727 (3·1)	564 (3·1)	371 (4·6)	655 (4·1)	421 (3·7)	6,464 (3·2)

Results are reported as number of individuals (percentage) for categorical variables and mean coefficients and standard deviations for continuous variables.

ESM Table 4. Baseline characteristics for men across child to adult body size categories.										
**Characteristic in male individuals**	**ChildLow**	**ChildAverage**	**ChildHigh**	**ChildLow**	**ChildAverage**	**ChildHigh**	**ChildLow**	**ChildAverage**	**ChildHigh**	**Total**
	**AdultLow**			**AdultAverage**			**AdultHigh**			
**No. of participants at baseline**	25,41	27,466	3,992	26,161	46,07	11,768	5,297	10,463	6,198	162,825
**Age (years)**	56·2 (8·1)	56·9 (8·0)	55·9 (7·8)	57·0 (8·1)	57·0 (8·2)	56·1 (8·1)	56·9 (8·0)	56·8 (7·9)	55·2 (8·0)	56·7 (8·1)
**Educational attainment (university degree) (%)**	11,001 (43·6)	10,990 (40·3)	1,879 (47·4)	7,861 (30·3)	14,658 (32·1)	4,242 (36·3)	1,038 (19·8)	2,536 (24·5)	1,804 (29·4)	56,009 (34·7)
**Townsend deprivation index**	-1·6 (3·0)	-1·5 (3·0)	-1·6 (3·0)	-1·6 (2·9)	-1·7 (2·8)	-1·6 (3·0)	-0·7 (3·2)	-1·2 (3·1)	-1·1 (3·1)	-1·6 (3·0)
**Height (cm)**	177·3 (7·0)	176·0 (6·4)	176·7 (6·6)	176·4 (7·0)	175·7 (6·5)	176·1 (6·7)	175·5 (7·1)	175·2 (6·6)	175·8 (6·7)	176·1 (6·7)
**Body mass index (kg/m^2)**	23·5 (1·7)	23·9 (1·5)	24·0 (1·5)	28·2 (1·6)	28·4 (1·6)	28·8 (1·7)	34·9 (3·2)	34·8 (3·0)	35·7 (3·8)	27·7 (4·1)
**Waist-to-hip ratio**	0·89 (0·1)	0·89 (0·1)	0·89 (0·1)	0·95 (0·1)	0·94 (0·1)	0·95 (0·1)	1·0 (0·1)	0·99 (0·1)	1·0 (0·1)	0·93 (0·1)
**Body fat percentage (%)**	20·5 (4·5)	20·0 (4·4)	20·7 (4·5)	26·5 (3·7)	25·9 (3·7)	26·7 (3·7)	32·9 (3·7)	32·3 (3·8)	33·2 (4·0)	25·0 (5·7)
**Glucose levels (mmol/L)**	4·9 (0·8)	4·9 (0·8)	4·9 (0·8)	5·1 (1·0)	5·0 (0·9)	5·1 (1·1)	5·4 (1·5)	5·4 (1·5)	5·4 (1·5)	5·1 (1·0)
**Glycated haemoglobin (HbA1c) (mmol/mol)**	34·6 (4·6)	34·4 (4·1)	34·2 (4·1)	35·7 (5·6)	35·3 (5·2)	35·3 (5·6)	38·5 (8·2)	37·8 (8·0)	37·7 (8·2)	35·4 (5·6)
**Glycated haemoglobin (HbA1c) (%)**	5·3 (0·4)	5·3 (0·4)	5·3 (0·4)	5·4 (0·5)	5·4 (0·5)	5·4 (0·5)	5·7 (0·8)	5·6 (0·7)	5·6 (0·8)	5·4 (0·5)
**Systolic blood pressure (mm/Hg)**	139·5 (18·4)	139·6 (18·6)	138·1 (18·5)	145·2 (17·9)	144·7 (18·0)	143·3 (18·1)	147·6 (18·1)	147·6 (17·7)	146·6 (18·3)	143·2 (18·4)
**Cholesterol levels (mmol/L)**	5·6 (1·0)	5·6 (1·0)	5·5 (1·0)	5·7 (1·1)	5·7 (1·1)	5·6 (1·1)	5·4 (1·1)	5·5 (1·1)	5·4 (1·1)	5·6 (1·1)
**LDL cholesterol (mmol/L)**	3·5 (0·8)	3·5 (0·8)	3·4 (0·8)	3·6 (0·9)	3·6 (0·8)	3·6 (0·8)	3·5 (0·9)	3·5 (0·8)	3·5 (0·8)	3·6 (0·8)
**HDL cholesterol (mmol/L)**	1·4 (0·3)	1·4 (0·3)	1·5 (0·4)	1·2 (0·3)	1·3 (0·3)	1·3 (0·3)	1·1 (0·2)	1·1 (0·2)	1·1 (0·2)	1·3 (0·3)
**Triglycerides (mmol/L)**	1·7 (0·9)	1·5 (0·9)	1·5 (0·8)	2·2 (1·2)	2·1 (1·2)	2·0 (1·2)	2·5 (1·3)	2·4 (1·3)	2·4 (1·3)	2·0 (1·1)
**No. of incident incident type 2 diabetes (%)**	605 (2·4)	535 (1·9)	74 (1·9)	1,983 (7·6)	2,579 (5·6)	728 (6·2)	1,179 (22·3)	1,855 (17·7)	1,102 (17·8)	10,640 (6·5)
**No. of incident incident cardiovascular disease (%)**	1,405 (5·5)	1,519 (5·5)	211 (5·3)	1,833 (7·0)	3,183 (6·9)	795 (6·8)	487 (9·2)	911 (8·7)	512 (8·3)	10,856 (6·7)

Results are reported as number of individuals (percentage) for categorical variables and mean coefficients and standard deviations for continuous variables.

ESM Table 5. Body mass index mean and standard deviation across child body size groups by						
	**Women**			**Men**		
**Age tertile**	**ChilLow**	**ChildAverage**	**ChildHigh**	**ChilLow**	**ChildAverage**	**ChildHigh**
**First**	25.24 (4.65)	26.22 (4.79)	29.35 (6.30)	26.54 (3.95)	27.64 (3.86)	30.08 (4.99)
**Second**	26.15 (4.64)	26.78 (4.73)	29.25 (5.88)	26.79 (3.93)	27.83 (3.89)	29.90 (4.77)
**Third**	26.57 (4.47)	26.89 (4.48)	29.06 (5.41)	26.89 (3.80)	27.60 (3.66)	29.48 (4.28)

ESM Fig. 1. Included individuals for the present analysis. Flowchart showing included individuals in the current analysis.

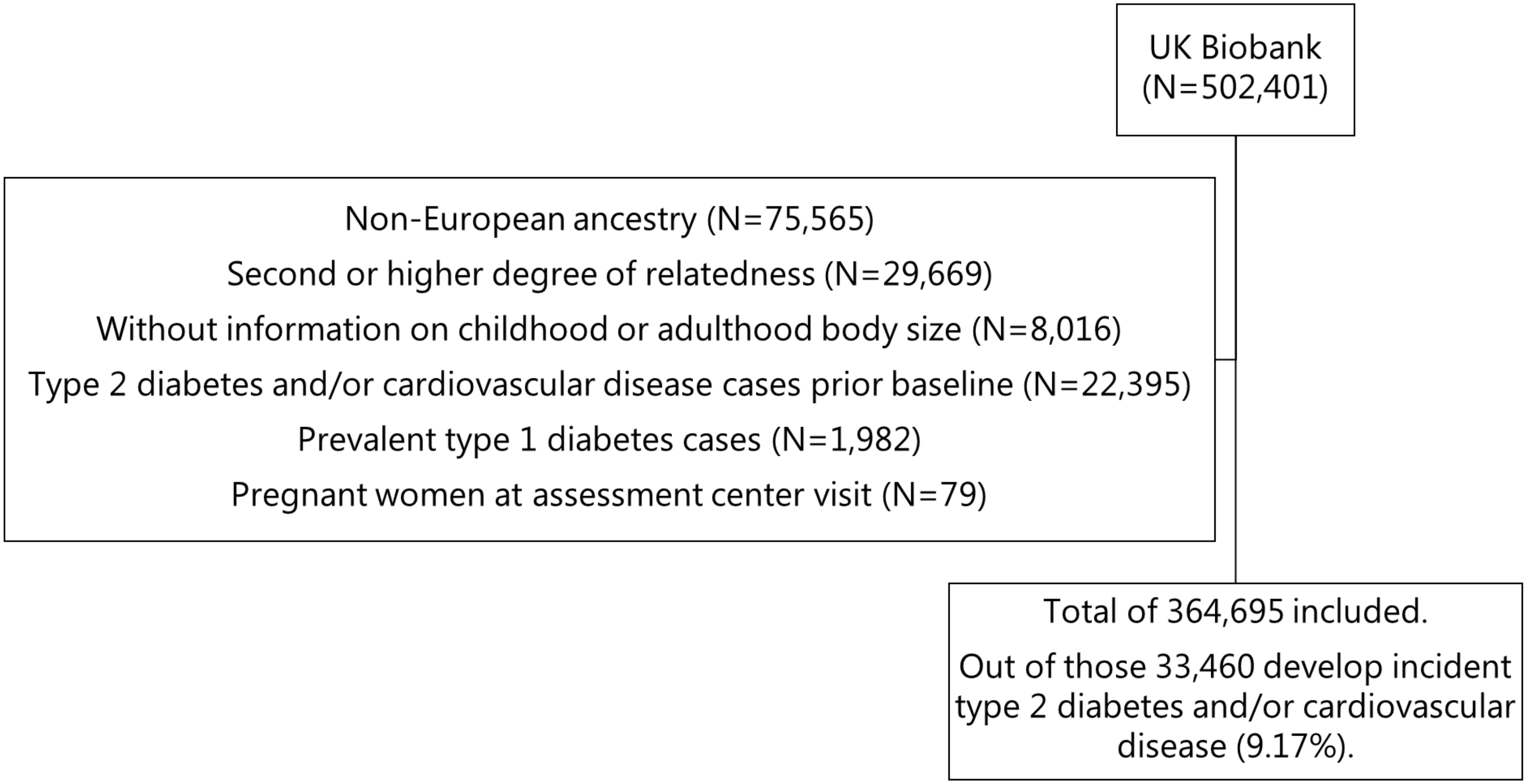

ESM Fig. 2. Definition and proportions for body size categories from childhood to adulthood across sexes.

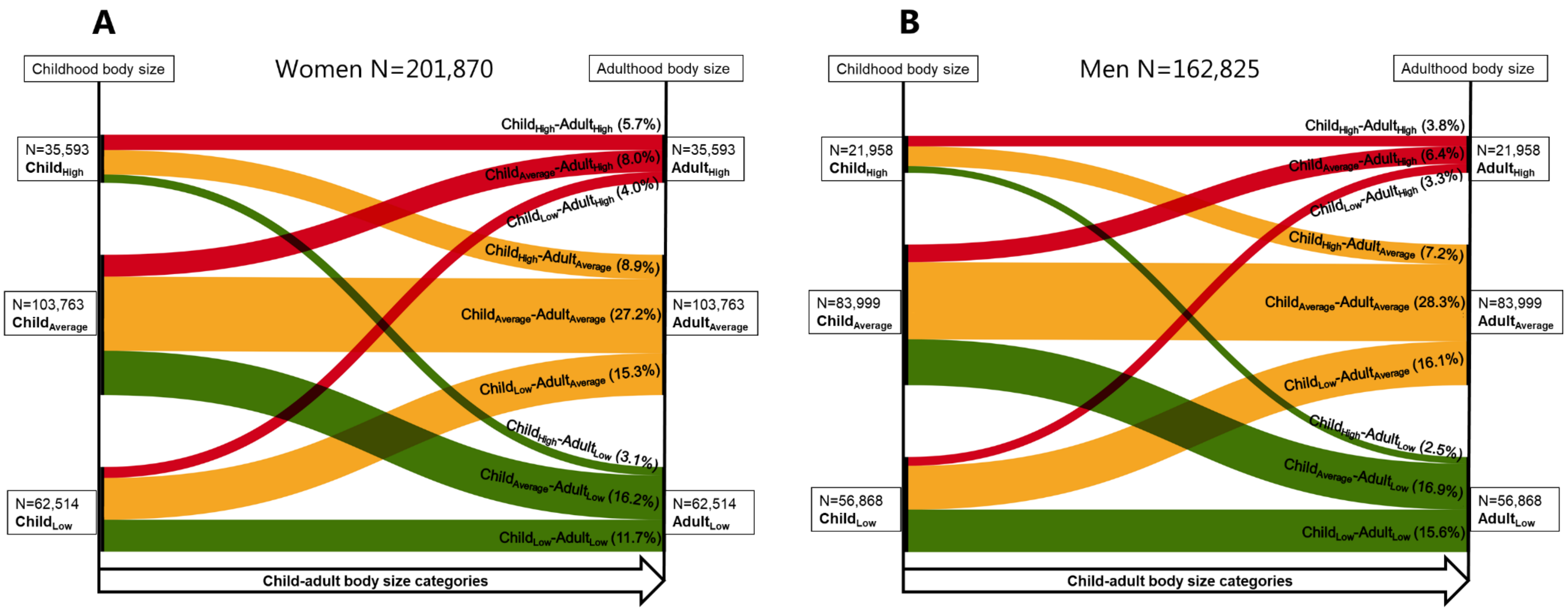

ESM Fig. 3. Type 2 diabetes risk across child and adult body size groups separately. The top panels are the Kaplan–Meier estimates of incidence disease (A-B), and the bottom panels are adjusted hazard ratios for disease risk (C-D). Cox regressions were adjusted for age, sex, Townsend deprivation index, and assessment centre.

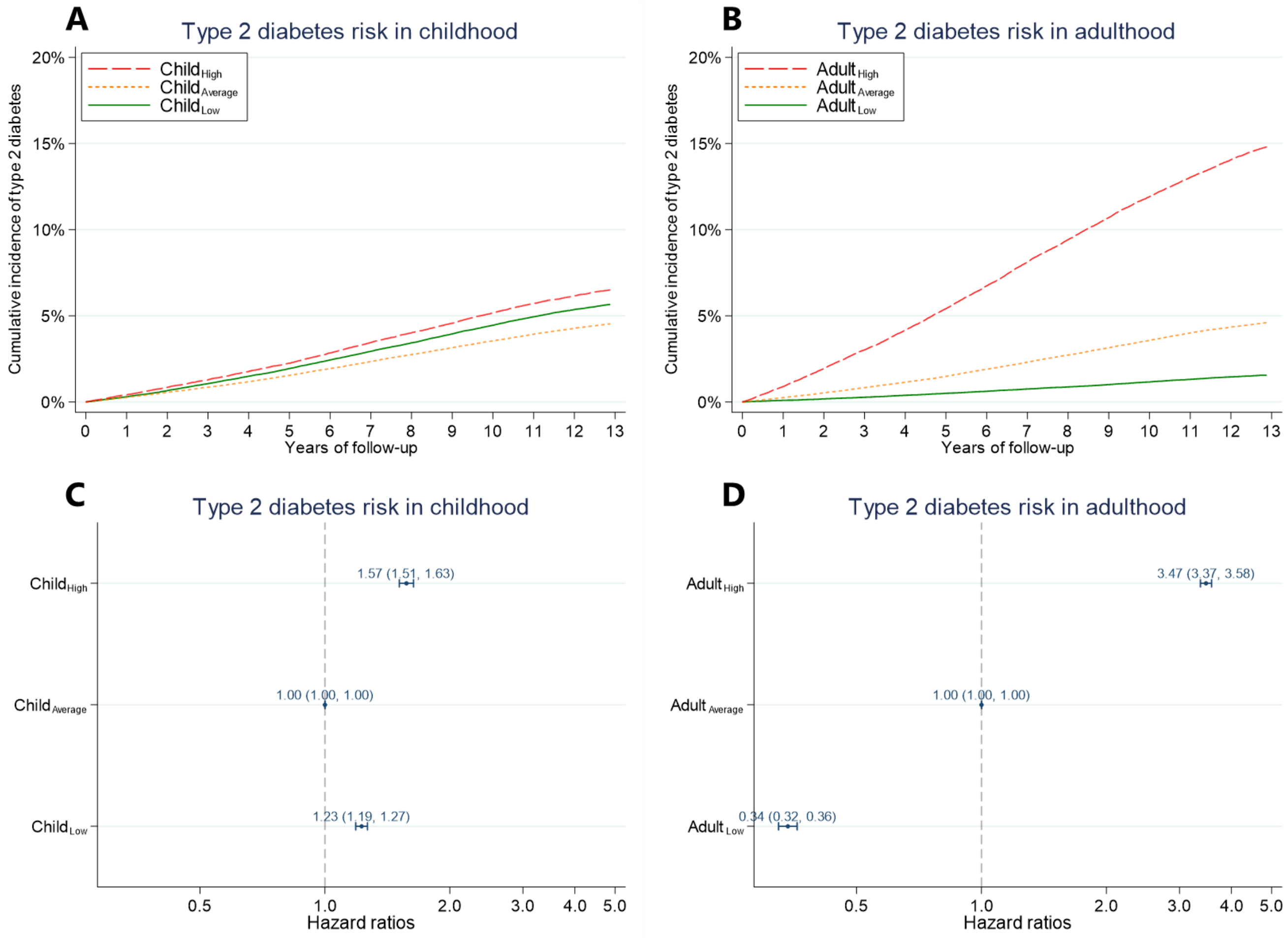

ESM Fig. 4. Cardiovascular risk across child and adult body size groups separately. The top panels are the Kaplan–Meier estimates of incidence disease (A-B), and the bottom panels are adjusted hazard ratios for disease risk (C-D). Cox regressions were adjusted for age, sex, Townsend deprivation index, and assessment centre.

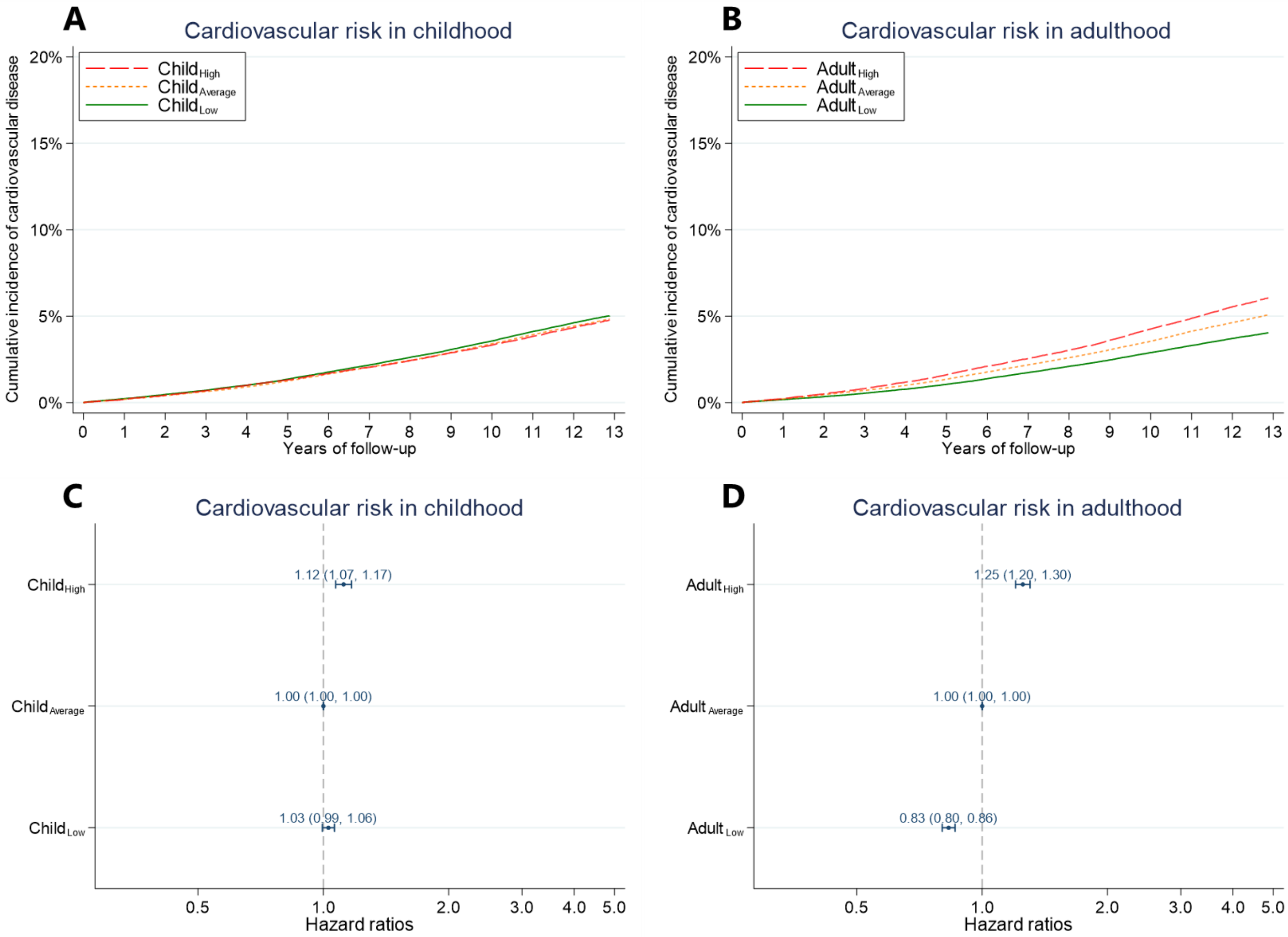

ESM Fig. 5. Sex-stratified type 2 diabetes and cardiovascular disease incidence by child-adult body size categories. Shown are the Kaplan–Meier estimates of the incidence of disease across body size change categories.

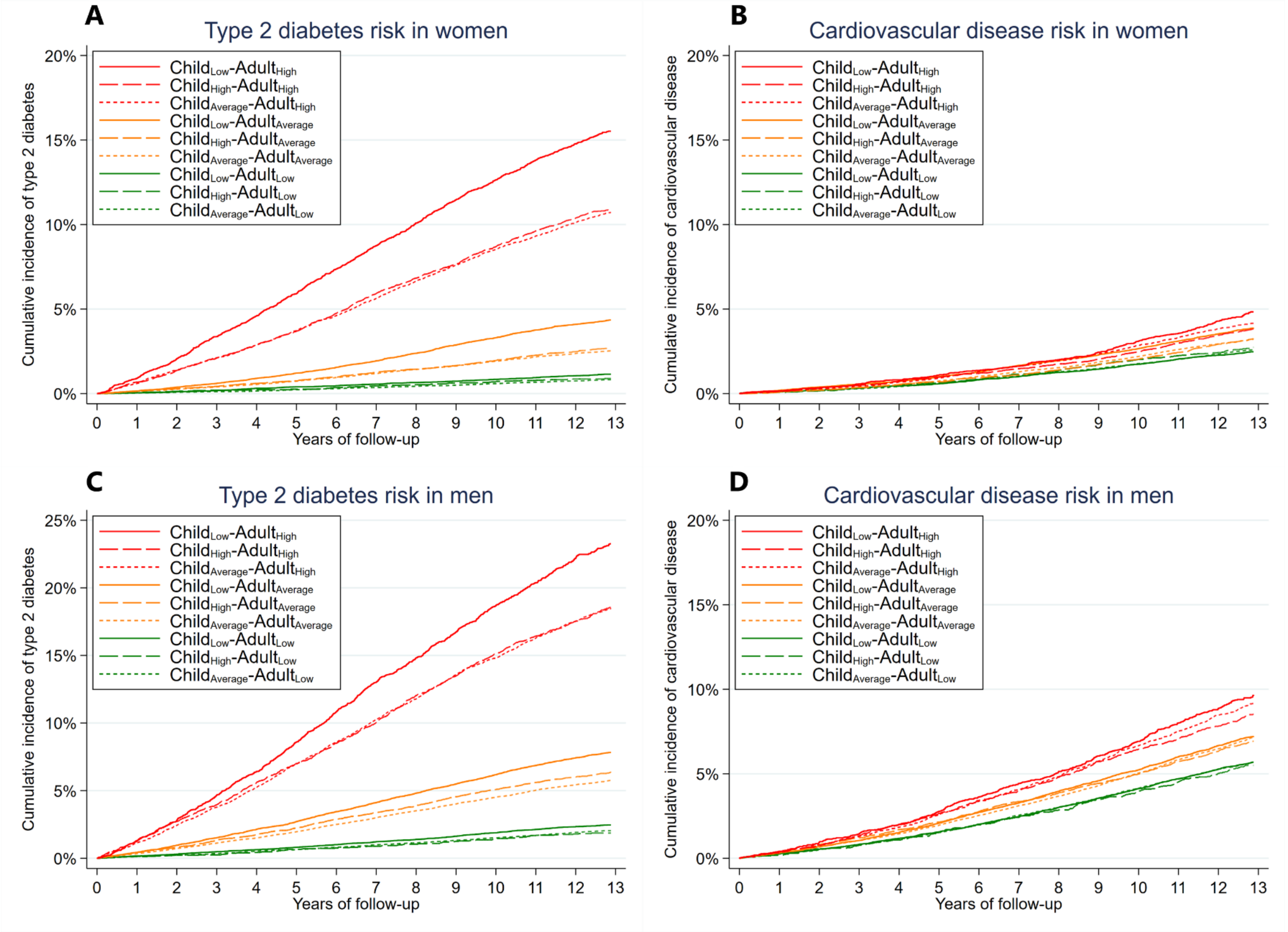

ESM Fig. 6. Sex-stratified risk of type 2 diabetes and cardiovascular disease across childhood-adulthood body size categories. Shown are adjusted hazard ratios for disease risk across childhood-adulthood body size categories. In these comparisons, individuals in the Child$_{Average}$-Adult$_{Average}$ served as the reference group. Cox regression was adjusted for age, educational attainment, Townsend deprivation index, and assessment centre. Bars indicate 95% confidence intervals.

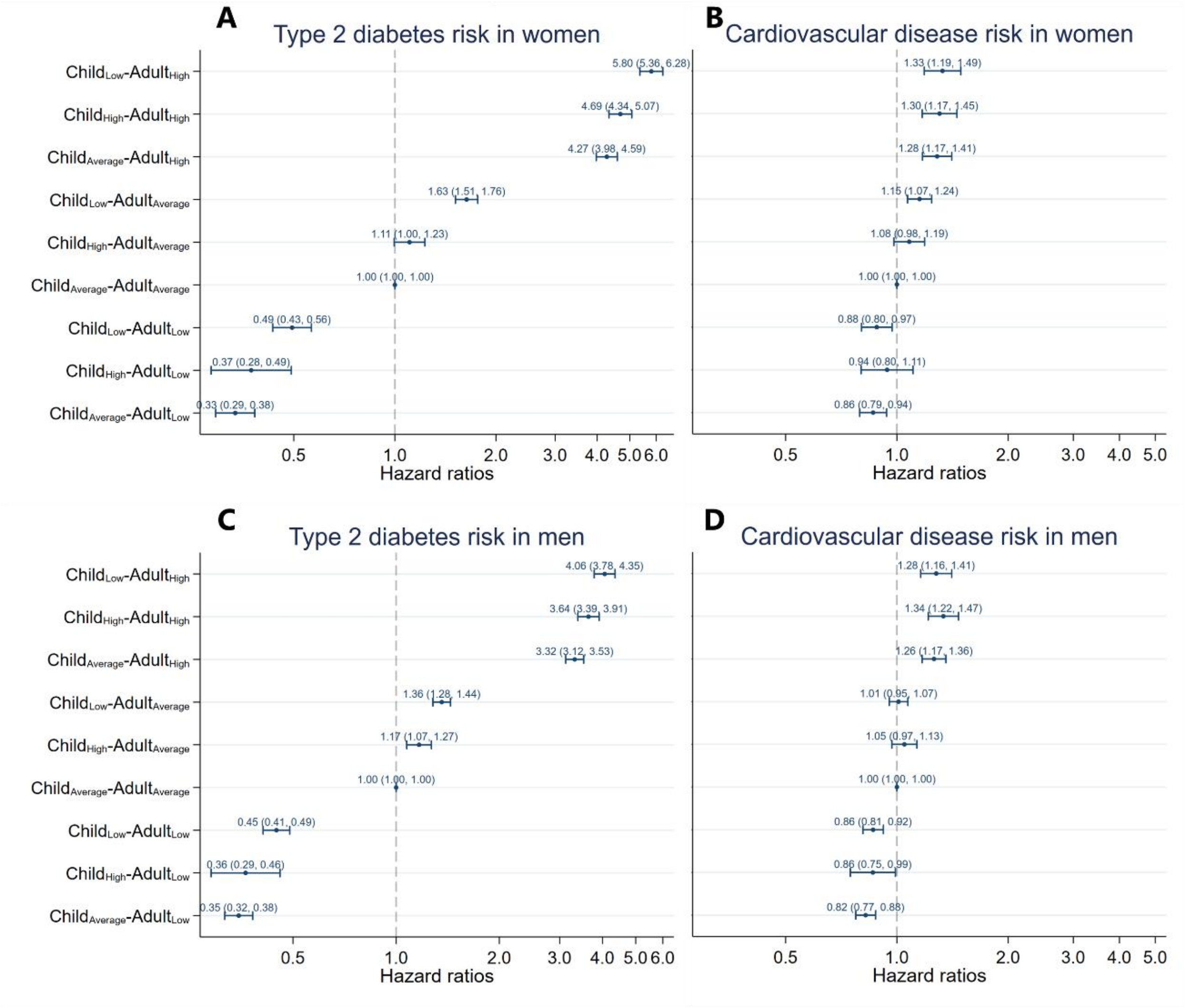

ESM Fig. 7. Myocardial infarction and stroke incidence by child-adult body size categories. Shown are the Kaplan–Meier estimates of the incidence of disease across body size change groups for combined and sex-stratified analysis.

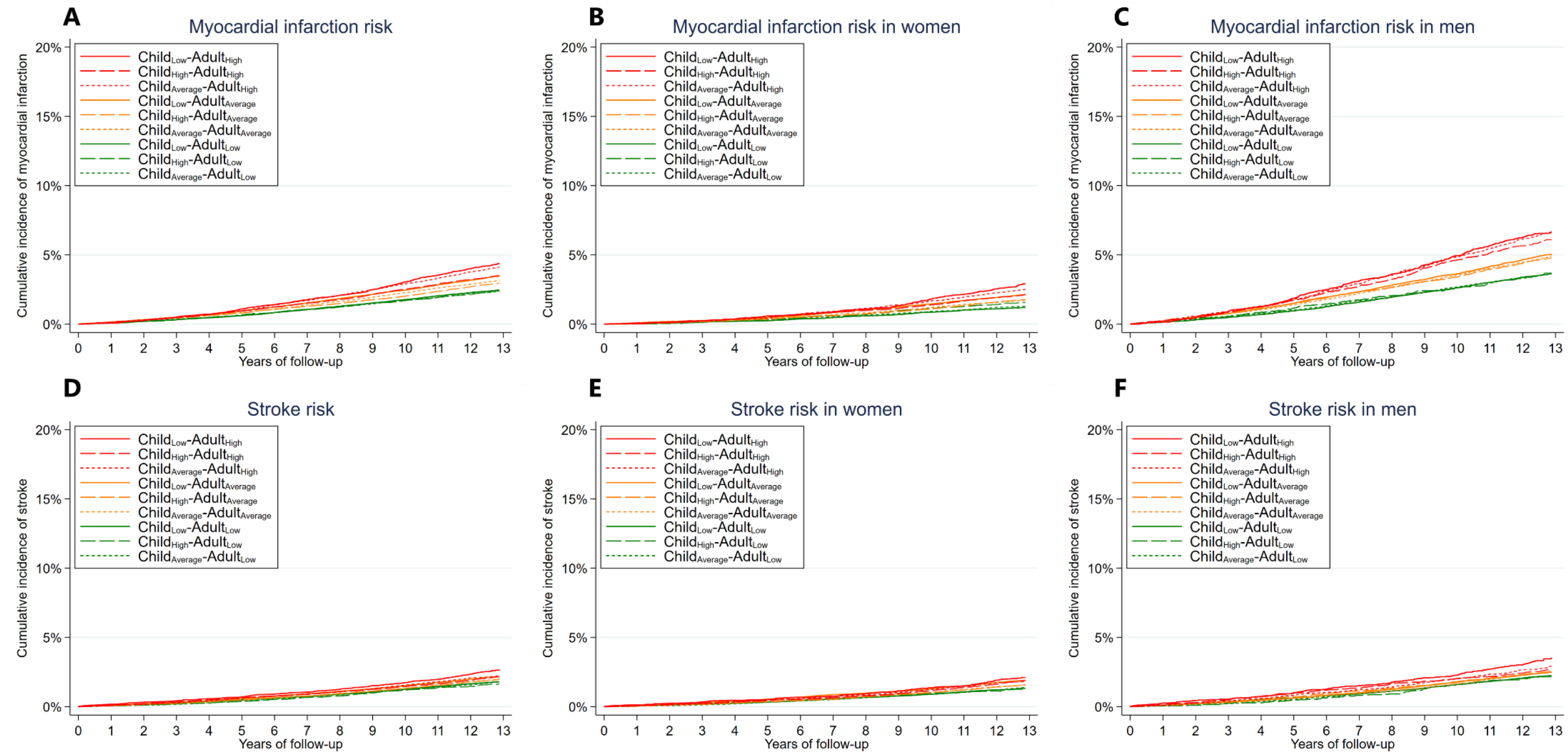

ESM Fig. 8. Risk of myocardial infarction and stroke by child-adult body size categories. Shown are adjusted hazard ratios for disease risk across childhood-adulthood body size categories for combined and sex-stratified analysis. In these comparisons, individuals in the $\text{Child}_{\text{Average}}$-$\text{Adult}_{\text{Average}}$ served as the reference group. Cox regression was adjusted for age, sex, educational attainment, Townsend deprivation index, and assessment centre. Bars indicate 95% confidence intervals.

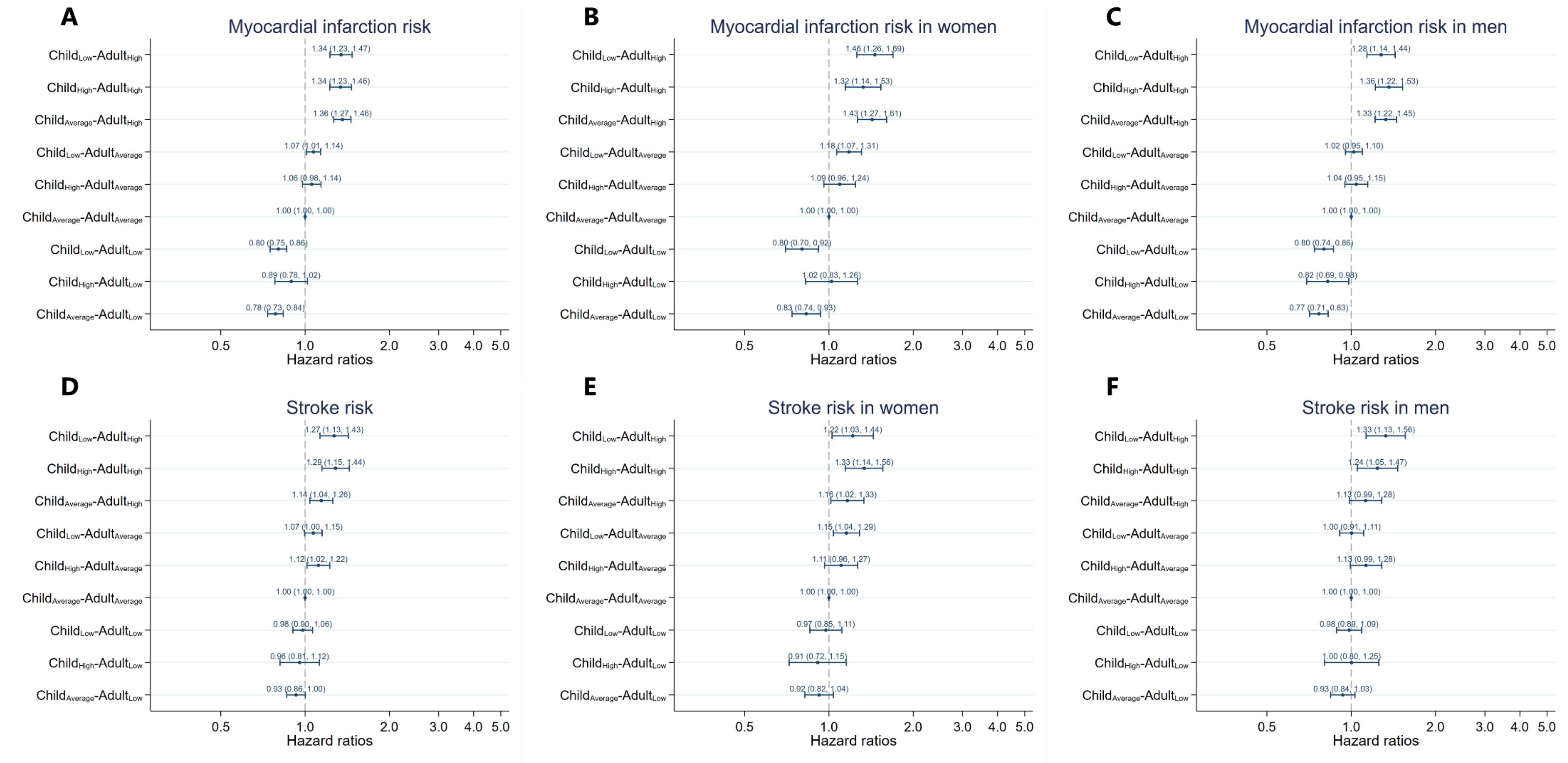

ESM Fig. 9. Odds ratios for type 2 diabetes and cardiovascular risk, including prevalent cases across child to adult body size categories. Panels A, B, and C show odd ratios for type 2 diabetes, and Panels D, E, and F show odds ratios for cardiovascular disease. Logistic regression models were adjusted for age, sex, educational attainment, Townsend deprivation index, and assessment centre. Bars indicate 95% confidence intervals.

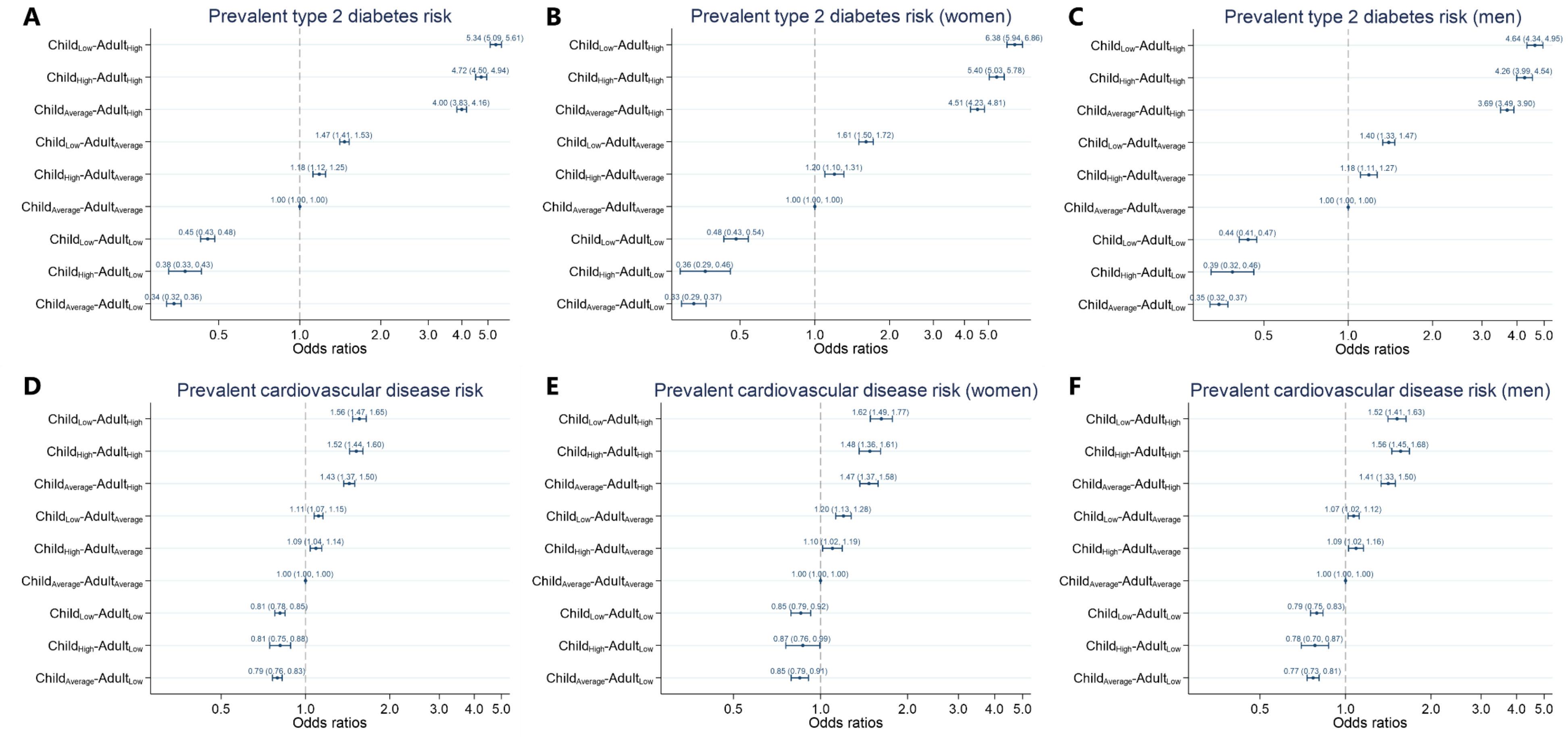

ESM Fig. 10. Risk of type 2 diabetes and cardiovascular disease across child to adult body size categories with and without accounting for birth weight. Hazard ratios for disease risk across child-adult body size categories for combined and sex-stratified analysis (women/men). Type 2 diabetes is shown in the panels A and C, and cardiovascular disease in panels B and D. Models were adjusted for the same covariates as the main model, including age, sex, Townsend deprivation index, and assessment centre (A and B). Birth weight was added to the model on top of the covariates above (C and D). In these comparisons, individuals in the $\text{Child}_{\text{Average}}$-$\text{Adult}_{\text{Average}}$ served as the reference group. Bars indicate 95% confidence intervals.

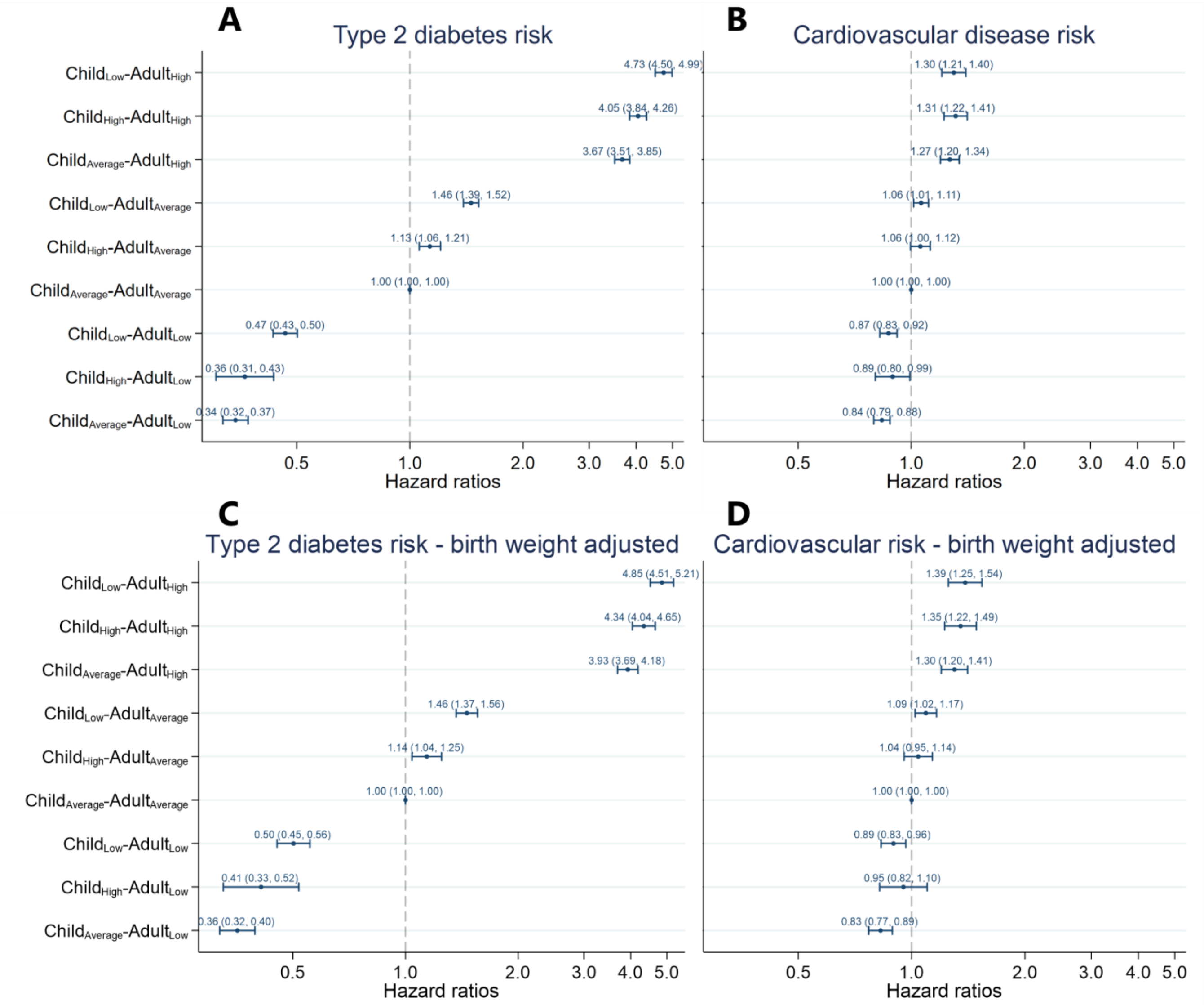

ESM Fig. 11. Risk of type 2 diabetes and cardiovascular disease by child-adult body size categories further adjusted. Shown are adjusted hazard ratios for disease risk across childhood-adulthood body size categories for combined and sex-stratified analysis. In these comparisons, individuals in the Child$_{Average}$-Adult$_{Average}$ served as the reference group. Cox regressions were adjusted for age, sex, educational attainment, Townsend deprivation index, assessment centre, smoking status, dietary score, physical activity, sedentary time, sleep hours, family history of diabetes, and prevalent diseases (cancer and severe respiratory diseases). Bars indicate 95% confidence intervals.

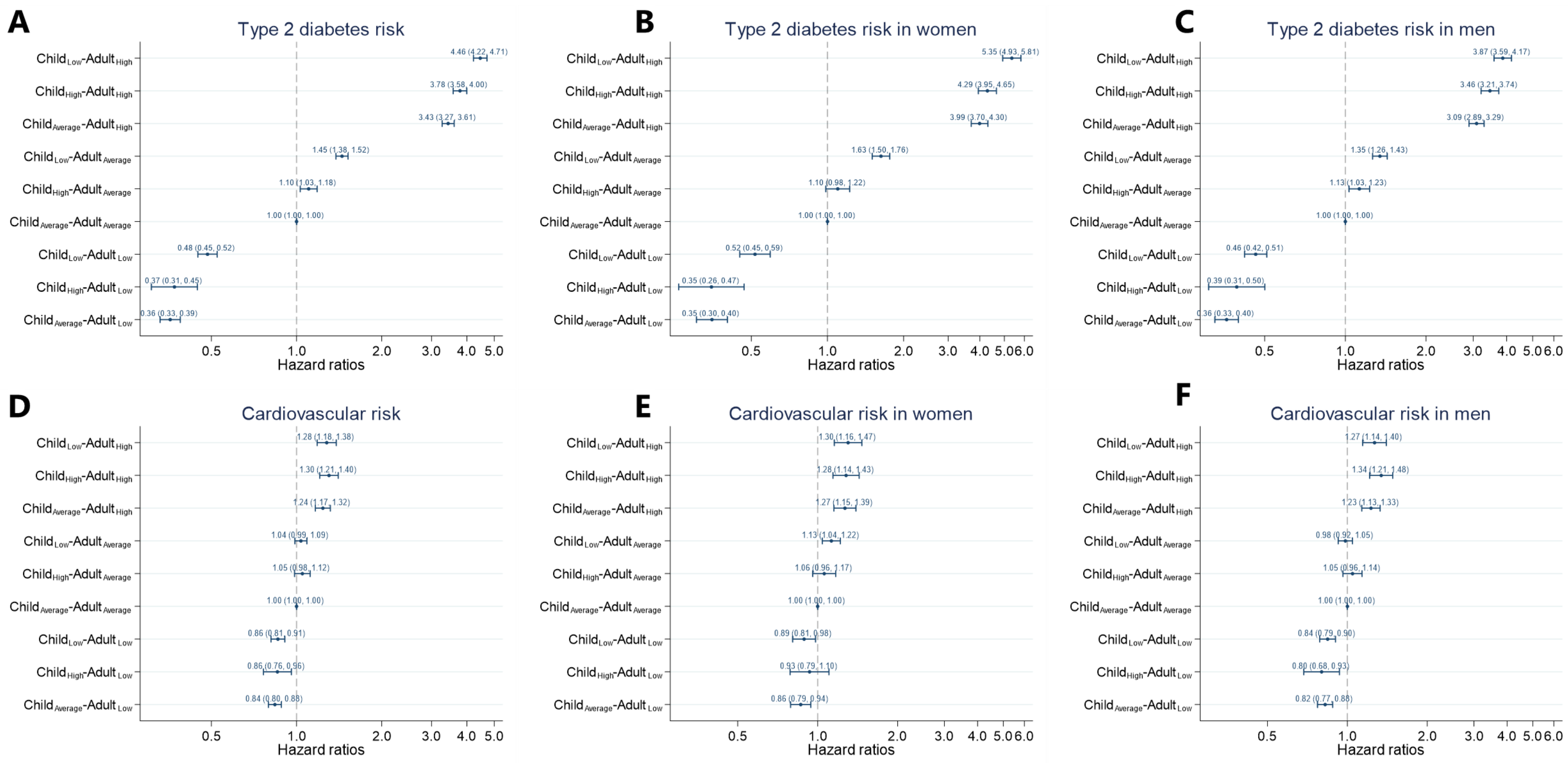

ESM Fig. 12. Risk of type 2 diabetes and cardiovascular disease by child-adult body size categories stratified by age tertiles. Shown are adjusted hazard ratios for disease risk across childhood-adulthood body size categories combined within each age tertile. In these comparisons, individuals in the $\text{Child}_{\text{Average}}$-$\text{Adult}_{\text{Average}}$ served as the reference group. Cox regression analyses were adjusted for age, sex, educational attainment, Townsend deprivation index, and assessment centre, the same as in the primary analysis. Bars indicate 95% confidence intervals.

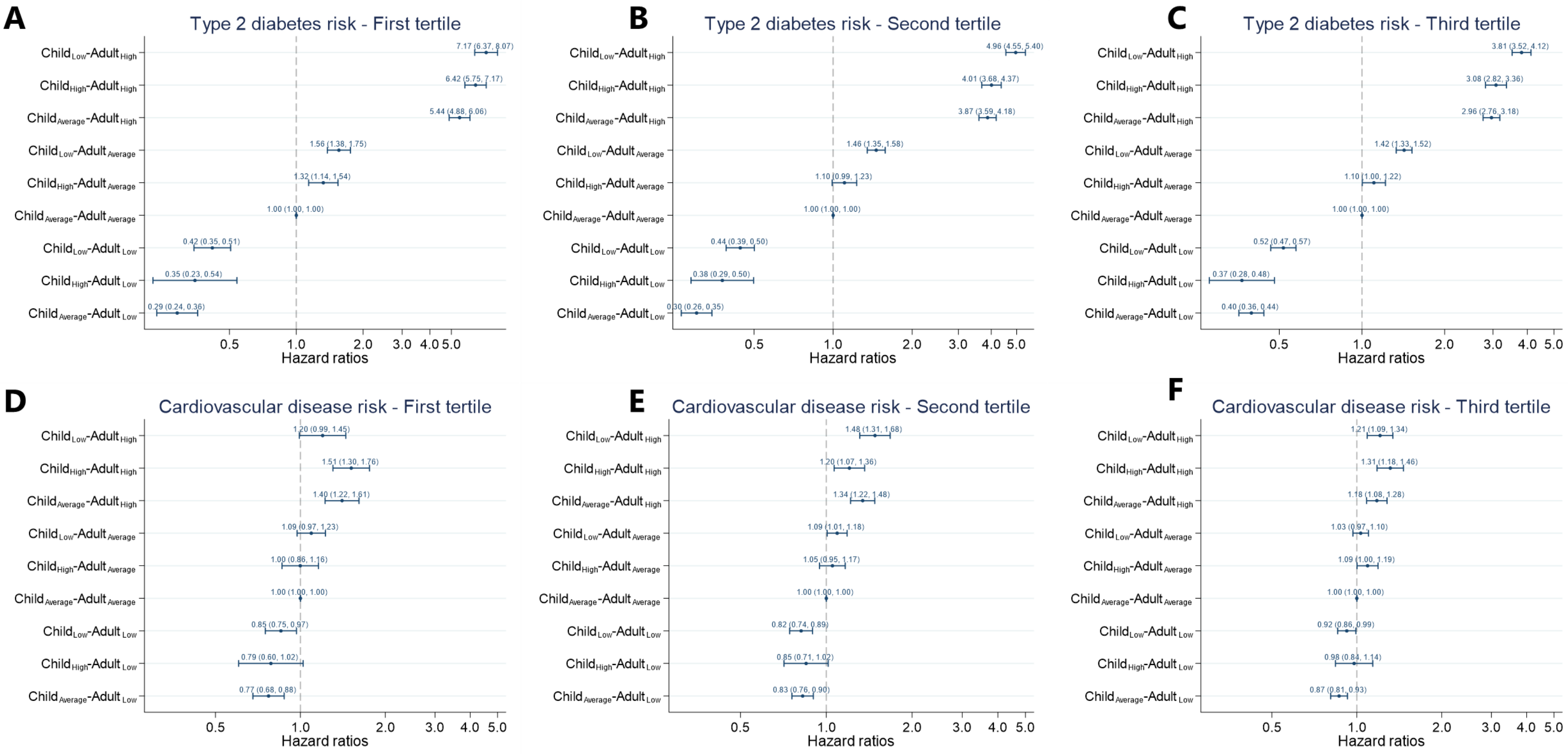

ESM Fig. 13. Disease risk across genetic risk tertiles for adult obesity for Child$_{Low}$-Adult$_{High}$ and Child$_{High}$-Adult$_{High}$ categories. Hazard ratios for disease risk are reported across tertiles of polygenic scores for adult obesity (high, medium, and low). Type 2 diabetes is shown on the left panel, and cardiovascular disease on the right. Models were adjusted for the same covariates as the main model, including age, sex, Townsend deprivation index, and assessment centre. In these comparisons, individuals in the Child$_{Average}$-Adult$_{Average}$ served as the reference group. Bars indicate 95% confidence intervals.

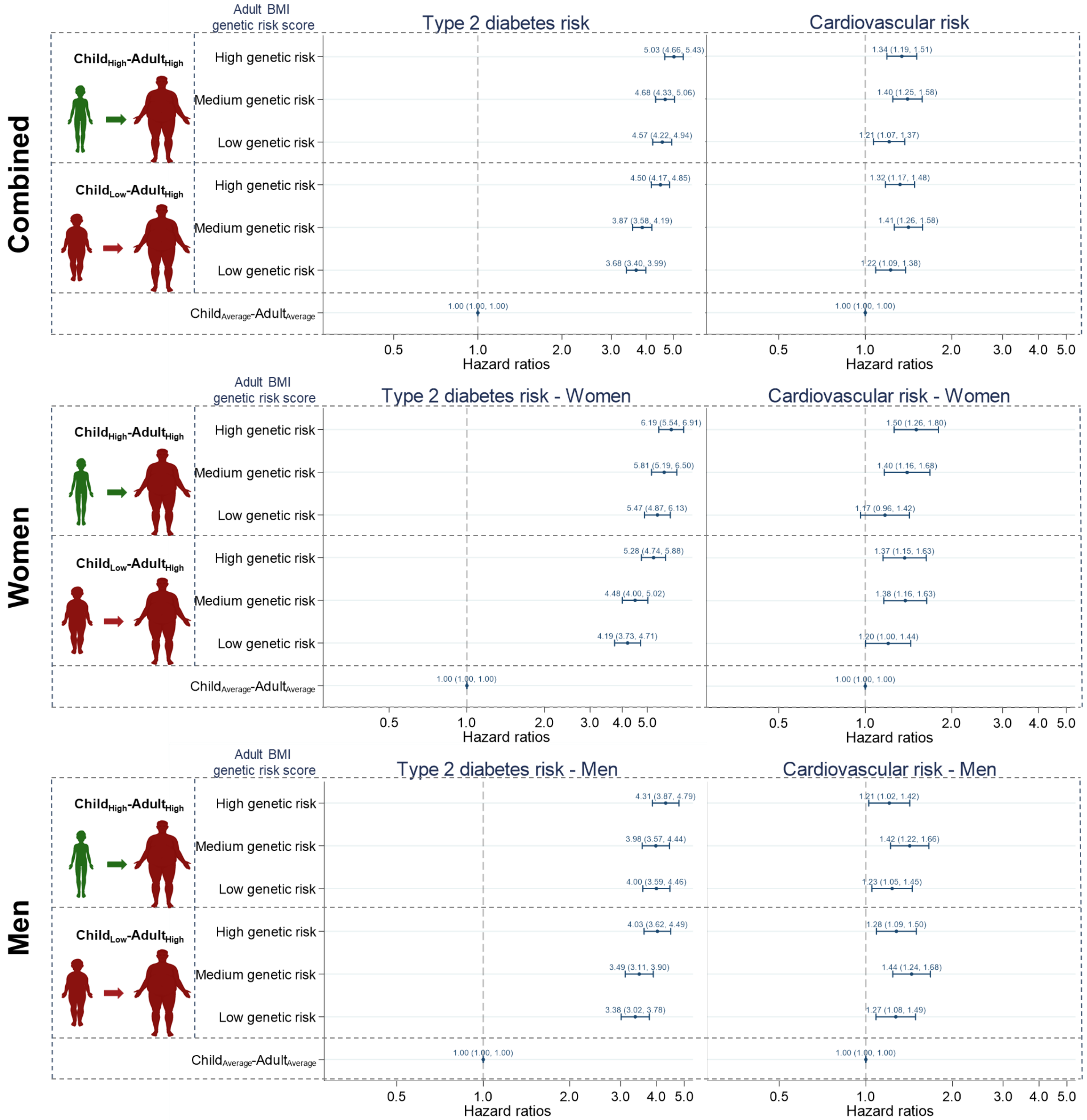

Supplement: Supplementary file 1 — Supplementary file1 (PDF 3.52 MB) [file 125_2023_6058_MOESM1_ESM.pdf]
